# Factors associated with extensions and withdrawals from *stricto sensu* graduate programs in Brazil during the COVID-19 pandemic: emphasis on mental health*

**DOI:** 10.1590/1518-8345.7988.4753

**Published:** 2026-01-19

**Authors:** Letuza Ribeiro Teixeira, Adriana Inocenti Miasso, Marina Aleixo Diniz Rezende, Nayara Paula Fernandes Martins Molina, Vanessa da Silva Carvalho Vila

**Affiliations:** 1 Pontifícia Universidade Católica de Goiás, Goiânia, GO, Brazil. Pontifícia Universidade Católica de Goiás GO Goiânia Brazil; 2 Universidade de São Paulo, Escola de Enfermagem de Ribeirão Preto, PAHO/WHO Collaborating Centre for Nursing Research Development, Ribeirão Preto, SP, Brazil. Universidade de São Paulo Escola de Enfermagem de Ribeirão Preto PAHO/WHO Collaborating Centre for Nursing Research Development SP Ribeirão Preto Brazil; 3 Scholarship holder at the Conselho Nacional de Desenvolvimento Científico e Tecnológico (CNPq), Brazil Scholarship holder at the Conselho Nacional de Desenvolvimento Científico e Tecnológico Brazil; 4 Scholarship holder at the Coordenação de Aperfeiçoamento de Pessoal de Nível Superior (CAPES), Brazil. Scholarship holder at the Coordenação de Aperfeiçoamento de Pessoal de Nível Superior Brazil

**Keywords:** Graduate Education, Mental Health, COVID-19, Universities, Teaching, Risk Factors

## Abstract

to analyze the factors associated with extension of the completion deadline and the course withdrawals by Brazilian students enrolled in *stricto sensu* graduate programs during the COVID-19 pandemic, with emphasis on mental health.

cross-sectional analytical study conducted with 5,286 graduate students enrolled in 2022. Data were collected through an electronic form that contained sociodemographic, academic, and health information, including a history of mental disorders. Descriptive and inferential statistical analyses were performed using logistic regression through R software.

the study found that 38% of participants extended their courses and 4.8% withdrew from enrollment. Extension was associated with pursuing a doctorate, studying Engineering or Biological Sciences, reporting training difficulties, and presenting mental health problems. Participation in remote classes and a previous diagnosis of hypertension emerged as protective factors. Withdrawal was associated with having children, studying Engineering, facing pandemic difficulties, presenting mental health problems, and having a history of academic leave. Being a scholarship holder was a protective factor, reducing the chances of withdrawal by 50%.

the findings indicate that extensions and withdrawals are related to academic, personal and health factors, highlighting the need for institutional policies that promote comprehensive support, with special attention to mental health and retention in graduate programs.

## Highlights

**(1)** 38% of graduate students extended their courses and 4.8% withdrew. **(2)** Academic difficulties and mental health impacted extensions and withdrawals. **(3)** Being a scholarship holder reduced the chance of withdrawing by 50%. **(4)** Remote classes were a protective factor against course extension. **(5)** Educational policies should promote academic support and mental well-being.

## Introduction

Over its 50-year existence, the Brazilian National Graduate System has established itself as a reference for advanced training in Latin America, playing an essential role in preparing academics and professionals for the country’s scientific, technological, and innovative advancements^(1-4)^. However, the COVID-19 pandemic imposed significant challenges on *stricto sensu* graduate students by amplifying structural inequalities and impacting mental health^(5-9)^.

A recent study indicated that 63.5% of Brazilian graduate students presented common mental disorders (CMD) during the pandemic, particularly women, young people and master’s students, especially those facing financial difficulties and academic delays^(5)^. Globally, researchers experienced elevated stress and anxiety due to economic insecurity, loss of employment opportunities and social isolation, reinforcing the need for institutional support^(6,10-12)^.

In Brazil, restricted access to laboratories, delays in research schedules, and the transition to emergency remote teaching emerged as critical barriers to continuing academic training, intensifying feelings of overload and loneliness among students^(5,13-14)^. The pressure for productivity in an uncertain scenario intensified psychological distress, often requiring temporary leave from academic activities^(6,11)^.

Added to this scenario were the impacts of financial difficulties, which worsened during the pandemic and were associated with increased depressive symptoms, providing further evidence of the importance of economic stability for maintaining students’ mental health and the need for specific interventions to support vulnerable individuals during crises^(3-7)^. Research also identified that students in *stricto sensu* graduate programs faced significant repercussions in their personal and academic lives, particularly due to the demands of adapting to emergency remote teaching and the need to cope with complex social, economic, and psychological problems^(11,15)^.

Given this scenario, many resorted to deadline extensions and enrollment withdrawals. In Brazil, these instruments are regulated by higher education institutions and follow standards established by the *Coordenação de Aperfeiçoamento de Pessoal de Nível Superior* (CAPES). Deadline extension allows for extending the maximum time stipulated for course completion, while withdrawal involves the temporary suspension of the student’s academic activities, maintaining their institutional affiliation.

Both instruments are essential for ensuring the continuity of academic training and providing the necessary support for students to overcome adversities without compromising teaching rigor and quality. In the pandemic context, the relevance of such strategies was reiterated through recognition and response to the complexity of challenges abruptly imposed on students’ trajectories^(15)^.

In this context, this study aimed to analyze the factors associated with deadline extensions and course withdrawals among Brazilian students enrolled in stricto sensu graduate programs during the COVID-19 pandemic, with an emphasis on mental health. This analysis aims to provide support for policies and strategies that ensure students’ mental health and academic continuity, especially during crisis periods.

## Method

### Study type and location

Cross-sectional analytical study following the Strengthening the Reporting of Observational Studies in Epidemiology (STROBE) guidelines^(16)^. The research was conducted virtually across the entire Brazilian territory using the Research Electronic Data Capture (REDCap) platform. Data collection occurred between May and July 2022.

### Population and sample

The study population consisted of students enrolled in *stricto sensu* graduate programs in Brazil in 2022. According to data from CAPES, in 2019, Brazil had 388,629 students enrolled in 4,565 graduate programs across 498 higher education institutions (HEIs), encompassing academic and professional master’s and doctoral degrees^(17)^.

Sample size calculation, considering the entire national territory, was based on an estimated prevalence of 50%, precision of 1.5%, and a 95% confidence interval, resulting in a minimum required sample of 4,222 participants. It is important to note that, in the absence of specific information about outcome prevalence, as is the case for graduate students, a calculation with 50% prevalence is recommended, as it ensures the sample size is sufficient for any observed prevalence value^(18)^.

The study included students enrolled in academic and professional *stricto sensu* graduate programs, aged 18 or older, of both sexes, residing in Brazil with internet access. Those who did not fully complete the data collection form were excluded. The study reached a total of 5,334 responses. For the deadline extension analysis, 5,245 valid responses to the specific question for this outcome were considered. For the enrollment withdrawal analysis, 5,286 valid responses to the respective questions were included. The two outcomes were analyzed independently and participants who reported experiencing both events were included in both analyses according to their individual responses to each variable.

### Study variables

The dependent variables analyzed were the occurrence of deadline extension and enrollment withdrawal. Deadline extension was defined as a self-reported formal request to extend the period originally stipulated for course completion. In contrast, enrollment withdrawal was characterized by a self-reported temporary and formal suspension of academic activities by the student.

Independent variables were organized into three main categories: sociodemographic, academic, and health history and mental disorders. Sociodemographic variables included age range, gender, sexual orientation, marital status, presence and number of children, monthly individual income, household composition, race/ethnicity and religion.

Academic variables addressed the type of *stricto sensu* graduate course (academic or professional), geographic region of the course, major field of knowledge, scholarship status, simultaneous work and study, weekly hours dedicated to studies, remote class attendance, perception of equivalence between remote and in-person classes, pandemic impact on academic training, quality of internet access for educational activities and satisfaction with digital teaching.

Variables related to health history and mental disorders encompassed multiple aspects, including fear of contracting or transmitting the coronavirus, COVID-19 immunization status (including the number of doses received) and membership in high-risk groups. The use of medications without a medical prescription during the pandemic was also assessed, including those for COVID-19 protection, as well as access to health services over the past year, with an emphasis on medical and psychological care.

Specific COVID-19-related issues were investigated, such as positive test results, need for hospitalization, positive cases among household members, and use of continuous medications, including psychotropic drugs. Additionally, smoking history with duration and daily cigarette consumption, self-medication, episodes of psychological distress during the pandemic, previous diagnoses of mental disorders and need for leave from academic activities due to psychological issues were also considered.

In the mental health field, self-reports of previous mental disorder diagnoses and during the pandemic were included, as well as psychotropic drug use, initiation or continuation of these medications during the pandemic, their prescription or absence and psychological care before and during the pandemic.

### Data collection instruments

A structured online questionnaire was used covering sociodemographic, academic, and health and mental disorder history data, developed by the leading project researchers with content validation. The Self-Reporting Questionnaire (SRQ-20) was also used, proposed by the World Health Organization (WHO) for screening common mental disorders (CMD) and validated in Brazil for use in the general population^(19)^.

The SRQ-20 comprises 20 questions distributed across four categories: somatic complaints, depressive and anxious mood, loss of vital energy, and depressive thoughts. The questions address physical symptoms (4 questions) and emotional symptoms (16 questions), with binary responses (yes/no). Each positive response adds one point, generating a total score ranging from zero to 20, with higher scores indicating a greater probability of non-psychotic mental disorders (NPMD). For this study, validated cut-off points were adopted: eight or more positive responses for women and six or more for men indicated suspected CMD.

### Data collection

Data collection was conducted in a structured and comprehensive manner. Initially, emails were sent to the 4,592 *stricto sensu* graduate programs in Brazil to request that they forward an invitation letter to regularly enrolled students. Program adherence was voluntary, and those who agreed to participate forwarded the recruitment email to their respective students.

The invitation letter presented detailed information about the study, including its objectives, relevance, and procedures, along with a link to the Informed Consent Form (ICF). After reading and accepting the ICF, participants were redirected to the online form, developed and made available on the REDCap platform.

Data collection was conducted entirely virtually through the REDCap platform, which ensures high standards of security, accessibility, and efficiency in information storage and processing. The use of this platform guarantees participant anonymity and data integrity throughout all research stages, promoting reliability and the protection of collected information. Information was stored in an encrypted form, with password authentication and restricted access to the team responsible, to ensure data confidentiality in accordance with the ethical principles applicable to human research.

### Data treatment and analysis

Data were compiled and exported to an electronic spreadsheet using Excel® Windows. Independent double data entry and verification of errors and inconsistencies were performed. Statistical analyses were conducted using R software version 4.2.3, supported by the Vector Generalized Additive Models (VGAM) package.

Analysis was conducted in two main stages. First, bivariate analysis was performed to identify statistically significant associations (α = 0.05) between independent variables and outcomes (deadline extension and enrollment withdrawal). The Likelihood Ratio Test was used to assess the significance of each variable in the model inclusion and exclusion stages, allowing comparisons between adjusted models.

In the subsequent stage, the binary logistic regression model was used to identify factors associated with the investigated outcomes. This model was chosen for its suitability in analyzing categorical variables and binary outcomes, allowing for the estimation of Odds Ratios and their 95% confidence intervals (CI). The significance level adopted for analyses was 5% (α = 0.05).

For the composition of final regression models, only variables that showed a statistically significant association in bivariate analysis were included. The likelihood ratio test was then applied to assess the permanence of variables in the adjusted models. This strategy aimed to ensure parsimony and statistical robustness while avoiding overfitting. Although various clinical and academic conditions were initially considered, only those that remained statistically significant were included in multivariate analyses.

In this study, all factors statistically associated with outcomes (extension and withdrawal) were referred to as related factors. For interpretation purposes, those with an odds ratio (OR) > 1 were considered risk factors, and those with an OR < 1, protective factors.

### Ethical aspects

The study was reviewed and approved by the Research Ethics Committee, under the Certificate of Presentation for Ethical Consideration (CAAE) no.: 56048822.9.0000.5393 and opinion no. 5,384,965. All precepts established in Resolution 466/12 of the National Health Council were respected.

## Results

From the sample of 5,245 students from whom valid responses related to deadline extension were obtained, a prevalence of 38.7% was identified for extension requests during the COVID-19 pandemic. Extension was predominantly requested by students aged 18 to 39 years (38%), who were single (38.8%), had no children (39%), reported no income (46.2%) or religious affiliation (42.2%), identified as non-binary (40.5%), non-heterosexual (42.5%), identified as “other” racial categories (46.7%) and lived with two to three people (39.6%).

In the academic context, extension had higher prevalence among doctoral students (44.9%), especially in the Southeast Region (39.2%), in the fields of Biological Sciences (49.3%) and Engineering (46.6%), scholarship holders (39.7%), and those with unrelated employment (41.3%). Additionally, these graduate students did not need to travel for studies (39.7%), dedicated more than 40 hours to studies (42.0%), did not have remote classes (60.1%), considered remote classes inferior to in-person classes (40.1%), felt their training was impaired (45.2%) and expressed dissatisfaction with online teaching (48.2%).

In the health context related to students’ COVID-19, higher prevalence of extensions was found among those who expressed fear of contracting and/or transmitting COVID-19 (38.4%) and were part of the risk group including pre-pandemic smoking (45.7%), pregnant women (48.2%), those with arterial hypertension (23.2%) and kidney problems (50.9%).

Deadline extensions were also more frequent among those who used medications in general without medical prescription (40.9%), used medications to protect themselves from COVID-19 on their own (37.9%), self-reported positive COVID-19 tests (39.2%), lived with people who tested positive for COVID-19 and developed severe symptoms requiring hospitalization (47.8%) and died (47.7%).

Furthermore, among the participants who requested deadline extensions, many reported using health services three or more times in the past year (39.7%). Additionally, many received medical care during the pandemic (41.2%) and many reported mental health problems (45.3%).

Regarding mental health, there was high prevalence of deadline extension among those who reported not feeling psychologically well in the past year (38.8%); underwent psychological care before the pandemic (40.2%), especially for Bipolar Affective Disorder (BAD) (50.9%), Generalized Anxiety Disorder (GAD) (41.6%), and depression (38.3%).

Higher prevalence was also observed among participants who, during the pandemic, received care for mental disorders (44.3%), most commonly Post-Traumatic Stress Disorder (PTSD) (51.6%), BAD (51.1%) and Panic Disorder (PD) (50.2%), as well as those who had a family member with a mental disorder (39.9%), most frequently in spouses (43.7%). Additionally, extension was observed in those with mental disorder diagnoses (44.4%), diagnoses of PTSD (49.5%), BAD (48.4%), and PD (46.1%), those who reported not feeling psychologically well during the pandemic and needed leave (49.9%), students with positive CMD (42.9%), and those who used psychotropic drugs during the pandemic (50.8%), most frequently cognitive enhancers (56.2%), antidepressants (45.2%), and anxiolytics (43.3%).

The final logistic regression analysis model revealed risk and protective factors for graduate students who requested deadline extensions for *stricto sensu* graduate course completion (Table 1).

**Table 1-:** Final logistic regression analysis of factors associated with deadline extension for *stricto sensu*graduate course completion (N = 5,245). Ribeirão Preto, SP, Brazil, 2022

**Variables**	b*	**SE-** b ^†^	**Z-value** ^‡^	p ^§^	**OR** ^||^	**CI (95%)** ^¶^	
(Intercept)	-0.95	0.31	-3.03	0.0025			
Northeast Region	-0.22	0,13	-1.71	0.09	0.80	0.62	1.03
North Region	-0.24	0.21	-1.18	0.23	0.78	0.52	1.17
Southeast Region	0.02	0.10	0.20	0.84	1.02	0.83	1.26
South Region	-0.04	0.14	-0.30	0.76	0.96	0.72	1.27
Doctorate	0.38	0.07	5.45	<0.0010	1.47	1.28	1.69
Engineering Course	0.40	0.14	2.97	0.0030	1.50	1.15	1.96
Biological Sciences Course	0.52	0.11	4.52	<0.0010	1.67	1.34	2.09
Having remote classes	-0.94	0.29	-3.24	0.0012	0.39	0.22	0.68
Perception of training impairment	0.76	0.08	9.39	<0.0010	2.14	1.83	2.51
COVID risk group - hypertensive	-0.96	0.35	-2.72	0.0066	0.38	0.18	0.74
Need for psychological care	0.35	0.07	4.74	<0.0010	1.41	1.22	1.63
Need for activity leave	0.51	0.08	6.78	<0.0010	1.68	1.44	1.95
Use of cognitive-enhancing drugs	0.63	0.21	2.96	0.0031	1.88	1.24	2.87
Positive for common mental disorder	0.27	0.08	3.41	˂0.0016	1.31	1.12	1.54

*b = Unstandardized regression coefficient; ^†^SE-b = Standard error; ^‡^Z-value = Standardized regression coefficient; ^§^p = Probability associated with statistical test; ^||^OR = Odds Ratio; ^¶^CI = Confidence interval

For the course withdrawal analysis, 5,286 valid responses were considered. Of this total, 254 participants (4.8%) reported having withdrawn from enrollment during the pandemic, constituting positive outcome cases. The highest percentage was observed among students aged 60 or older (11.9%), who had partners (5.7%), one child or fewer (8.8%) and self-declared their racial identity as “other” (8.5%).

Withdrawal was higher among graduate students in the North (6.7%), Central-West (6.0%) and South (4.9%) regions. Within the CAPES major field of Engineering (7.5%), non-scholarship holders predominated (6.1%), who had formal employment (6.5%), dedicated less than 40 weekly hours to studies (4.9%), did not have remote classes (8.0%), reported impairments in their training (5.7%) and dissatisfaction with online teaching (6.0%).

For course withdrawal, prevalence was higher among graduate students belonging to the COVID-19 risk group (8.0%), mainly those with kidney problems (18.9%) and pregnant women (12.3%). It was also higher in individuals who required hospitalization for COVID-19 (15.2%), lived with people who tested positive for the disease (5.4%), presented severe symptoms requiring hospitalization (9.8%) and sought health services three or more times in the past year (5.4%).

Withdrawal was also more frequent among those who received medical care during the pandemic (6.4%), especially in groups with vascular problems (10.1%), gastrointestinal issues (7.8%) and mental health problems (7.7%).

Still in the mental health context, higher prevalences for withdrawal were observed among students who reported not feeling psychologically well in the past year (5.0%), who sought psychological care before the pandemic with reports of BAD diagnoses (17.0%) and depression (7.2%) and who underwent psychological care before the pandemic (5.5%) and were already being treated for schizophrenia prior to the pandemic (33.3%).

Moreover, withdrawal was also more observed among those who underwent psychological care during the pandemic (6.6%), with diagnoses of PTSD (17.4%), BAD (17.0%) and PD (10.5%), who had a family member with a mental disorder diagnosis (5.8%), especially children (14.1%) and spouses (8.6%); those who already had mental disorder diagnoses (8.5%), notably PTSD (16.2%), BAD (12.9%), and depression (9.9%); those who needed to take leave from their activities due to not feeling well (8.7%); and those with positive CMD (5.9%), among those who used psychotropic drugs before the pandemic (7.5%) and those who began using them during this period (10.6%). Examples of the most commonly used psychotropic drugs included sedatives (11.5%), antipsychotics (10.7%) and cognitive enhancers (10.2%).

The final logistic regression analysis model revealed risk and protective factors for graduate students who requested enrollment withdrawal from the *stricto sensu* graduate course (Table 2).

**Table 2-:** Final logistic regression model of factors associated with *stricto sensu* graduate course withdrawal (N= 5,286). Ribeirão Preto, SP, Brazil, 2022

**Variables**	b*	**SE-** b ^†^	**Z-value** ^‡^	p ^§^	**OR** ^||^	**CI (95%)** ^¶^	
(Intercept)	-4.99	0.32	-15.67	p<0.001			
Northeast Region	-0.11	0.28	-0.40	0.69	0.90	0.52	1.54
North Region	0.69	0.36	1.93	0.05	2.00	0.96	3.97
Southeast Region	0.17	0.22	0.78	0.44	1.19	0.78	1.84
South Region	-0.03	0.31	-0.08	0.94	0.98	0.52	1.78
Number of children/one	1.03	0.19	5.33	p<0.001	2.81	1.91	4.08
Number of children/two or more	0.60	0.24	2.49	0.013	1.83	1.12	2.90
Engineering	0.75	0.24	3.14	0.0017	2.11	1.30	3.31
Being a scholarship holder	-0.69	0.16	-4.23	p<0.001	0.50	0.36	0.69
Perception of training impairment	0.65	0.20	3.26	0.0011	1.91	1.31	2.85
Perception of training impairment	0.88	0.16	5.40	p<0.001	2.41	1.76	3.33
Need for activity leave	1.24	0.18	6.95	p<0.001	3.44	2.44	4,91
Positive for CMD**	0.31	0.21	1.50	0.13	1.36	0.92	2.07

*b = Unstandardized regression coefficient; †SE-b = Standard error; ‡Z-value = Standardized regression coefficient; ^§^p = Probability associated with statistical test; ^||^OR = Odds Ratio; ^¶^CI = Confidence interval; **CMD = Common Mental Disorder

## Discussion

The results of this study indicate that deadline extension and enrollment withdrawal in *stricto sensu* graduate programs in Brazil were directly associated with academic, financial, and mental health challenges during the investigated period. The COVID-19 pandemic aggravated these factors by exposing structural vulnerabilities in higher education^(20)^ and hindering study continuity across different fields of knowledge^(6-7,11-12,21-23)^. The health crisis imposed abrupt transformations on academic institutions, requiring adaptations in teaching models and student support.

Among the main risk factors related to deadline extension were enrollment in doctoral programs, studying Engineering or Biological Sciences, perception of training impairments, and presenting mental health problems. Enrollment withdrawal, in turn, was related to studying Engineering, perception of training impairments intensified by the pandemic, mental disorder diagnoses and the need for academic leave.

Thus, while deadline extension reflects an attempt at academic continuity despite adversities, withdrawal represents a more significant interruption of the educational path, generally associated with more severe conditions of emotional, institutional, or health compromise. Although both outcomes are linked to student vulnerability, they require distinct institutional responses, tailored to the degree of compromise and context-specific characteristics experienced by students.

The rapid transition to remote teaching revealed critical structural difficulties, such as a lack of technological infrastructure and digital exclusion of students with lower purchasing power^(3,7,14)^. Such obstacles unequally affected graduate programs, being particularly evident in fields that depend on practical activities, such as Engineering, where migration to remote teaching significantly limited the development of experimental research, especially those requiring access to laboratories and field activities^(24)^.

Even before the pandemic, doctoral students already faced challenges in maintaining physical and mental well-being due to intense academic workload and lack of time for self-care^(9,15,25)^. With the pandemic, these difficulties were aggravated by the need to reconcile remote teaching, research continuity and family responsibilities, often without adequate financial or social support^(11-12,26-27)^.

Academic overload, uncertainties about the future, and personal losses intensified disorders such as anxiety and depression, highlighting the need for more effective institutional support^(5,15,28)^. High academic demands and productivity significantly affected graduate students’ mental health, who began presenting high stress levels with consequent compromise of academic retention and performance^(5,11)^. Furthermore, the difficulty of balancing academic, personal, and financial demands can be recognized as a critical obstacle to these students’ well-being, reinforcing the importance of institutional strategies to mitigate these impacts^(6,11-12)^.

The pandemic significantly worsened the scenario of psychological distress among students, especially those in socioeconomic vulnerability, due to social isolation and reduced face-to-face interactions^(3-4,7,24)^. The prevalence of common mental disorders (CMD) among Brazilian graduate students reached 63.5%, associated with factors such as academic difficulties, financial insecurity, deadline extensions, and temporary study leaves^(5,27)^. In response to experienced pressures, many resorted to using psychotropic drugs without medical supervision, highlighting the urgency of implementing institutional policies that strengthen mental health support and encourage safer and more effective coping strategies^(9,15,25)^.

Studies indicate that during the COVID-19 pandemic, there was a significant increase in anxiety and depression symptoms among university students, especially those in health fields. The prevalence of depressive symptoms varied between 31% and 38%, while anxiety symptoms reached about 41%^(29)^. Among graduate researchers, approximately 70% reported mild to severe psychological distress, despite higher levels of mental health literacy^(30)^. These findings underscore the importance of institutional policies to ensure continuous mental health care for this population, particularly given prolonged exposure to multiple academic and personal stressors^(25,27,29-30)^.

The presence of academic difficulties, intensified by the pandemic, migration to remote teaching and overload experienced by faculty and students during this period, may have contributed to students’ greater emotional vulnerability, highlighting the need for institutional strategies to strengthen academic support and mental health^(11,24)^. According to research, a positive relationship between advisor and graduate student can significantly reduce academic stress (β = -0.27, p < 0.01), with this effect partially mediated by self-efficacy (β = -0.14, p < 0.01) and moderated by psychological resilience (β = -0.10, p < 0.01)^(26)^. In this context, the quality of academic advising and the existence of a supportive environment prove fundamental for minimizing the negative impacts of graduate studies^(11,26)^ and reducing the need for extensions and withdrawals^(9,12)^.

Among participants who reported access to remote classes, a lower frequency of course extension was observed, justifying this result as acting as a protective factor in this subgroup, despite representing only 40% of the sample. Despite difficulties adapting to remote teaching, participation in virtual classes proved to be a protective factor against deadline extensions, possibly due to the flexibility provided by this mode of instruction. The ability to better manage time, access new learning opportunities, and increase productivity favored students’ academic continuity^(6,21-22,24)^.

Previous studies have already indicated that eliminating the need for physical commuting facilitates many graduate students’ retention in their courses by reducing logistical barriers and optimizing the time dedicated to academic activities^(4,24)^. Additionally, expanded access to courses and content previously restricted to specific locations, along with flexible study schedules, primarily benefited students balancing academic and professional activities, making teaching more accessible and adaptable to their realities^(11,24,26)^.

A noteworthy finding is that previous diagnosis of systemic arterial hypertension, although classified as a risk factor for COVID-19 complications, was identified in this study as a protective factor against course extension in *stricto sensu* graduate programs. A possible explanation for this result is that students with hypertension, being under continuous medical care, tend to show greater adherence to self-care practices, greater awareness about stress management, and better structuring of personal and academic routines, even facing pandemic-imposed adversities.

Although hypertension is generally associated with worse clinical outcomes^(31)^, it may also function as a marker of greater health vigilance, particularly in individuals with access to medical services and therapeutic support^(32)^. According to recent studies, factors such as physical activity, psychological resilience, and positive coping strategies can mitigate stress effects on the mental and academic health of hypertensive individuals^(31-32)^. Thus, the previous diagnosis may reflect not only a chronic condition but also a trajectory of adaptation and care that may have favored the continuity of academic activities during the pandemic period.

Nevertheless, this is an unusual finding that has been explored little in scientific literature. Therefore, complementary investigation in future studies is necessary to elucidate the mechanisms involved and assess whether this pattern is confirmed in other academic populations or institutional contexts.

The implications of this study underscore the urgent need for institutional policies that promote mental health and mitigate the effects of academic overload in graduate programs. Effective strategies include expanding psychological support, training advisors, academic flexibility, and strengthening student assistance to ensure more equitable conditions for student retention^(5,8-9)^. Furthermore, the importance of reformulating curricular matrices to make them more effective and flexible is highlighted, ensuring the sustainability of graduate programs and reducing the incidence of extensions and dropouts^(21)^.

Strengthening psychological support proved essential, involving expansion of university counseling services, creation of support groups, welcoming actions and academic mentoring programs. These initiatives may contribute to a healthier university environment and reduce the negative impacts of academic pressure on students^(21,25)^.

Although the relationship between advisor and student was not directly evaluated in this study, literature highlights its importance as a determining factor for academic success^(26-27)^. Advisor training, therefore, proves fundamental. Institutional strategies that encourage continuous faculty development, focusing on more humanized advising that is sensitive to students’ needs, can minimize the negative impacts of inadequate supervision. Implementation of institutional guidelines establishing good practices in academic advising directly contributes to a more productive, collaborative and less stressful environment^(26-27)^.

Academic flexibility should be encouraged to allow adjustments in deadlines for completing courses and dissertations, so students can overcome periods of greater difficulty without compromising their educational trajectory. Adapting assessments and promoting more flexible curricula make teaching more accessible and sustainable, consequently reducing rates of deadline extensions and enrollment withdrawals^(28)^.

Financial support and student assistance are essential to ensure the retention of graduate students. Expanding research scholarships, financial aid for transportation and housing, and creating student financing programs can minimize academically motivated dropouts due to economic difficulties. Financial security demonstrably reduces student vulnerability and improves their ability to face the challenges of graduate study^(5,21)^.

Furthermore, promoting well-being and healthy coping strategies should be encouraged through cultural, sports, and social activities that act as protective factors for mental health. Peer mentoring programs, encouragement of physical activity, and creation of social spaces within universities help reduce stress and strengthen social support networks^(26,28)^.

Finally, continuous monitoring of students’ mental health should be implemented through periodic institutional surveys to support the development of effective support policies. Developing anonymous platforms for reporting academic malpractices and harassment can contribute to a safer and more welcoming academic environment. Adopting institutional strategies that prioritize both academic excellence and student well-being is essential for a more sustainable and equitable educational system^(3,7,9,11,25,29-30)^.

Despite this study’s contributions, some limitations should be considered. Cross-sectional design prevents the determination of causality between the analyzed factors and academic outcomes, limiting identification to associations. Self-reported data collection may have generated response bias, especially on sensitive issues such as mental health and financial insecurity. Additionally, the absence of qualitative data restricts a deeper understanding of graduate students’ perceptions regarding institutional support and individual coping strategies.

Sample representativeness may not encompass all realities of Brazilian graduate education, indicating the need for future studies to address different institutional and regional contexts. Longitudinal research is crucial for evaluating the long-term effects of institutional policies on academic retention and students’ mental health. Additionally, qualitative approaches can deepen the analysis of the challenges faced and strategies used by students to deal with academic adversity^(25)^.

Given the identified challenges, educational institutions must invest in integrated strategies that promote mental health and strengthen graduate student retention. Expanding psychological support, humanized advisor training, academic flexibility, investment in student assistance, and promoting well-being should comprise a set of continuous and accessible actions, tailored to students’ sociocultural and digital diversity. Such measures not only reduce dropout rates and psychological distress but also strengthen the educational system, contributing to a more equitable, healthy path conducive to scientific and professional development.

## Conclusion

This study demonstrated that deadline extension and course withdrawal in *stricto sensu* graduate programs during the COVID-19 pandemic were influenced by risk and protective factors of academic, personal and psychosocial nature. Extension was mainly associated with enrollment in doctoral programs, the Engineering and Biological Sciences fields, perception of training impairments, and presence of mental disorders. In contrast, participation in remote classes and a previous diagnosis of hypertension acted as protective factors. However, limitations such as poor technological infrastructure and reduced social interaction posed significant challenges for many students.

Withdrawal was related to factors such as parenthood, difficulties experienced during the pandemic, history of leave, and mental health problems, while receiving a scholarship emerged as a protective factor. The results underscore the importance of mental health in academic retention and emphasize the need for institutional policies that prioritize psychosocial support, reduce socioeconomic inequalities, offer flexible educational pathways and foster more inclusive academic environments, particularly in crisis contexts.

## Data Availability

The dataset that supports the findings of this study is not publicly available.
