# Supplementary material for: Factors associated with extensions and withdrawals from stricto sensu graduate programs in Brazil during the COVID-19 pandemic: emphasis on mental health
Source: Rev Lat Am Enfermagem. 2026 Jan 19;34:e4753. doi: 10.1590/1518-8345.7988.4753 (PMC12854666; doi:10.1590/1518-8345.7988.4753)
Supplement: Translation

# Translation: Fatores associados às prorrogações e aos trancamentos da pós-graduação *stricto sensu* no Brasil na pandemia COVID-19: ênfase na saúde mental*

**DOI:** 10.1590/1518-8345.7988.4754

**Authors:** Letuza Ribeiro Teixeira, Adriana Inocenti Miasso, Marina Aleixo Diniz Rezende, Nayara Paula Fernandes Martins Molina, Vanessa da Silva Carvalho Vila

**Affiliations:** 1 Pontifícia Universidade Católica de Goiás, Goiânia, GO, Brasil. Pontifícia Universidade Católica de Goiás GO Goiânia Brasil; 2 Universidade de São Paulo, Escola de Enfermagem de Ribeirão Preto, Centro Colaborador da OPAS/OMS para o Desenvolvimento da Pesquisa em Enfermagem, Ribeirão Preto, SP, Brasil. Universidade de São Paulo Escola de Enfermagem de Ribeirão Preto Centro Colaborador da OPAS/OMS para o Desenvolvimento da Pesquisa em Enfermagem SP Ribeirão Preto Brasil; 3 Bolsista do Conselho Nacional de Desenvolvimento Científico e Tecnológico (CNPq), Brasil Bolsista do Conselho Nacional de Desenvolvimento Científico e Tecnológico Brasil; 4 Bolsista da Coordenação de Aperfeiçoamento de Pessoal de Nível Superior (CAPES), Brasil. Bolsista da Coordenação de Aperfeiçoamento de Pessoal de Nível Superior Brasil

**Keywords:** Educação de Pós-Graduação, Saúde Mental, COVID-19, Universidades, Ensino, Fatores de Risco

## Abstract

analisar os fatores associados à prorrogação de prazo de conclusão e ao trancamento de curso por discentes brasileiros matriculados em programas de pós-graduação *stricto sensu* no contexto da pandemia de COVID-19, com ênfase na saúde mental.

estudo transversal analítico, realizado com 5.286 pós-graduandos matriculados no ano de 2022. Os dados foram coletados por meio de formulário eletrônico com informações sociodemográficas, acadêmicas e de saúde, incluindo histórico de transtornos mentais. Empregou-se análise estatística descritiva e inferencial, com regressão logística, por meio do *software* R.

verificou-se que 38% dos participantes prorrogaram seus cursos e 4,8% trancaram a matrícula. A prorrogação esteve associada a cursar doutorado, Engenharias ou Ciências Biológicas, relatar dificuldades na formação e apresentar problemas de saúde mental. Participação em aulas remotas e diagnóstico prévio de hipertensão emergiram como fatores protetores. O trancamento associou-se a ter filhos, cursar Engenharia, enfrentar dificuldades na pandemia, apresentar problemas de saúde mental e histórico de afastamento acadêmico. Ser bolsista foi fator protetor, reduzindo em 50% as chances de trancamento.

os achados indicam que a prorrogação e o trancamento estão relacionados aos fatores acadêmicos, pessoais e de saúde, o que evidencia a necessidade de políticas institucionais promotoras de suporte integral, com atenção especial à saúde mental e à permanência na pós-graduação.

## Destaques

**(1)** 38% dos pós-graduandos prorrogaram o curso e 4,8% trancaram suas matrículas. **(2)** Dificuldades acadêmicas e saúde mental impactaram prorrogação e trancamento. **(3)** Ser bolsista reduziu em 50% a chance de trancamento da pós-graduação. **(4)** Aulas remotas foram fator protetor para a prorrogação do curso. **(5)** Políticas educacionais devem promover suporte acadêmico e bem-estar mental.

## Introdução

Ao longo de 50 anos de existência, o Sistema Nacional de Pós-Graduação brasileiro consolidou-se como referência em formação avançada na América Latina, com papel essencial na capacitação de acadêmicos e profissionais para o avanço científico, tecnológico e inovador do país^(1-4)^. No entanto, a pandemia de COVID-19 impôs desafios significativos aos estudantes de pós-graduação *stricto sensu*, por ampliar desigualdades estruturais e impactar na saúde mental^(5-9)^.

Estudo recente apontou que 63,5% dos pós-graduandos brasileiros apresentaram transtornos mentais comuns (TMC) durante a pandemia, sobretudo mulheres, jovens e estudantes de mestrado, especialmente aqueles que enfrentaram dificuldades financeiras e atrasos acadêmicos^(5)^. Em escala global, pesquisadores vivenciaram estresse e ansiedade elevados por insegurança financeira, perda de oportunidades de emprego e isolamento social, reforçando a necessidade de suporte institucional^(6,10-12)^.

No Brasil, a restrição de acesso aos laboratórios, os atrasos nos cronogramas de pesquisa e a transição para o ensino remoto emergencial configuraram-se como barreiras críticas à continuidade da formação acadêmica e potencializaram a sensação de sobrecarga e solidão entre os estudantes^(5,13-14)^. A pressão por produtividade em um cenário de incerteza intensificou o sofrimento psíquico, muitas vezes com necessidade de afastamento temporário das atividades acadêmicas^(6,11)^.

A esse cenário somaram-se os impactos das dificuldades financeiras, que se agravaram durante a pandemia e estiveram associadas ao aumento de sintomas depressivos, em mais uma evidência da importância da estabilidade econômica para a manutenção da saúde mental dos discentes e da necessidade de intervenções específicas para o apoio de indivíduos em situação de vulnerabilidade durante crises^(3-7)^. Pesquisas identificaram também que os discentes de programas de pós-graduação *stricto sensu* enfrentaram repercussões marcantes em suas vidas pessoais e acadêmicas, sobretudo pela exigência de adaptação ao ensino remoto emergencial e necessidade de enfrentamento de problemas sociais, econômicos e psicológicos complexos^(11,15)^.

Diante desse cenário, muitos recorreram à prorrogação de prazos e ao trancamento de matrícula. No Brasil, esses instrumentos são regulamentados pelas instituições de ensino superior e seguem as normativas estabelecidas pela Coordenação de Aperfeiçoamento de Pessoal de Nível Superior (CAPES). A prorrogação de prazo de conclusão permite ampliar o tempo máximo estipulado para a finalização do curso, enquanto o trancamento consiste na suspensão temporária das atividades acadêmicas do discente, mantendo seu vínculo com a instituição.

Ambos os instrumentos são essenciais para assegurar a continuidade da formação acadêmica e proporcionam o suporte necessário para que os discentes superem adversidades sem comprometer o rigor e a qualidade do ensino. No contexto pandêmico, a relevância de tais estratégias foi reiterada, pelo reconhecimento e resposta à complexidade dos desafios abruptamente impostos na trajetória dos discentes^(15)^.

Nesse contexto, o presente estudo teve como objetivo analisar os fatores associados à prorrogação de prazo de conclusão e ao trancamento de curso pelos discentes brasileiros matriculados em programas de pós-graduação *stricto sensu* no contexto da pandemia de COVID-19, com ênfase na saúde mental. Esta análise objetiva oferecer subsídios para políticas e estratégias que assegurem a saúde mental e a continuidade acadêmica dos discentes, especialmente em períodos de crise.

## Método

### Tipo e local de estudo

Estudo transversal analítico, conforme as diretrizes do *Strengthening the Reporting of Observational Studies in Epidemiology* (STROBE)^(16)^. A pesquisa foi realizada de forma virtual e abrangeu todo o território brasileiro, utilizando a plataforma *Research Electronic Data Capture* (REDCap). A coleta de dados ocorreu entre maio e julho de 2022.

### População e amostra

A população do estudo foi composta por discentes matriculados em programas de pós-graduação *stricto sensu* no Brasil em 2022. De acordo com dados da Coordenação de Aperfeiçoamento de Pessoal de Nível Superior (CAPES), em 2019, o Brasil contava com 388.629 discentes distribuídos em 4.565 programas de pós-graduação em 498 instituições de ensino superior (IES), abrangendo mestrados e doutorados acadêmicos e profissionais^(17)^.

O cálculo do tamanho amostral, considerando todo o território nacional, foi baseado em uma prevalência estimada de 50%, precisão de 1,5% e intervalo de confiança de 95%, o que resultou em uma amostra mínima necessária de 4.222 participantes. Importante destacar que, na ausência de informações específicas sobre a prevalência de desfechos, como é o caso dos discentes de pós-graduação, o cálculo com prevalência de 50% é recomendado, pois garante que o tamanho amostral seja suficiente para qualquer valor de prevalência observado^(18)^.

Foram incluídos no estudo discentes matriculados em programas de pós-graduação *stricto sensu* acadêmicos e profissionais, com idade mínima de 18 anos, de ambos os sexos, residentes no Brasil e com acesso à internet. Foram excluídos aqueles que não completaram integralmente o formulário de coleta de dados. O estudo alcançou um total de 5.334 respostas. Para a análise de prorrogação de prazo, foram consideradas válidas 5.245 respostas à pergunta específica para este desfecho. Já para a análise de trancamento de matrícula, foram incluídas 5.286 respostas válidas à respectiva questão. Os dois desfechos foram analisados de forma independente e os participantes que relataram ter vivenciado ambos os eventos foram incluídos nas duas análises, conforme suas respostas individuais a cada variável.

### Variáveis do estudo

As variáveis dependentes analisadas foram a ocorrência de prorrogação de prazo e de trancamento de matrícula. Definiu-se a prorrogação de prazo como o autorrelato de solicitação formal para estender o período originalmente estipulado para a conclusão do curso, enquanto o trancamento de matrícula foi caracterizado pelo autorrelato de suspensão temporária e formal das atividades acadêmicas por parte do discente.

As variáveis independentes foram organizadas em três categorias principais: sociodemográficas, acadêmicas e histórico de saúde e transtornos mentais. As variáveis sociodemográficas incluíram faixa etária, gênero, orientação sexual, situação conjugal, presença e quantidade de filhos, renda individual mensal, composição do domicílio, cor/raça e religião.

As variáveis acadêmicas abordaram o tipo de curso de pós-graduação *stricto sensu* (acadêmico ou profissional), a região geográfica do curso, a grande área do conhecimento, a condição de bolsista, a simultaneidade entre trabalho e estudos, a carga horária semanal dedicada aos estudos, a realização de aulas remotas, a percepção de equivalência entre aulas remotas e presenciais, o impacto da pandemia na formação acadêmica, a qualidade do acesso à internet para atividades acadêmicas e o grau de satisfação com o ensino digital.

As variáveis relacionadas ao histórico de saúde e aos transtornos mentais abrangeram múltiplos aspectos, como o medo de contrair ou transmitir o coronavírus, *status* de imunização contra a COVID-19 (incluindo o número de doses recebidas) e pertencimento aos grupos de risco. Também foi avaliado o uso de medicamentos sem prescrição médica durante a pandemia, inclusive daqueles para proteção contra a COVID-19 e o acesso aos serviços de saúde no último ano, com ênfase no acompanhamento médico e psicológico.

Foram investigadas questões específicas relacionadas à COVID-19, como a ocorrência de testes positivos, necessidade de internações, evolução de casos positivos entre conviventes e uso de medicamentos contínuos, incluindo psicofármacos. Adicionalmente, histórico de tabagismo, com tempo de consumo e quantidade de cigarros por dia, automedicação, episódios de mal-estar psicológico durante a pandemia, diagnósticos prévios de transtornos mentais e necessidade de afastamento das atividades acadêmicas por questões psicológicas também foram considerados.

No campo da saúde mental, foram incluídos autorrelatos de diagnósticos prévios de transtornos mentais e durante a pandemia, utilização de psicofármacos, início ou continuidade do uso desses medicamentos durante a pandemia, sua prescrição ou ausência, bem como acompanhamento psicológico antes e durante a pandemia.

### Instrumentos de coleta de dados

Utilizou-se um questionário *online* estruturado que contemplou dados sociodemográficos, acadêmicos, histórico de saúde e transtornos mentais elaborado pelos pesquisadores do projeto matriz e com validação de conteúdo. Também foi utilizado o *Self-Reporting Questionnaire* (SRQ-20), proposto pela Organização Mundial da Saúde (OMS) para rastreamento de transtornos mentais comuns (TMC) e validado no Brasil para o rastreamento de transtornos mentais comuns na população geral^(19)^.

O SRQ-20 é composto por 20 perguntas distribuídas em quatro categorias: queixas somáticas, humor depressivo e ansioso, perda de energia vital e pensamentos depressivos. As questões abordam sintomas físicos (4 questões) e emocionais (16 questões), com respostas binárias (sim/não). Cada resposta positiva soma um ponto, gerando uma pontuação total que varia de zero a 20, sendo que maiores pontuações indicam maior probabilidade de transtornos mentais não psicóticos (TMNP). Para este estudo, foram adotados os pontos de corte validados: oito ou mais respostas positivas para mulheres e seis ou mais para homens indicaram suspeita de TMC.

### Coleta de dados

A coleta de dados foi conduzida de maneira estruturada e abrangente. Inicialmente, e-mails foram enviados aos 4.592 programas de pós-graduação *stricto sensu* do Brasil, a fim de solicitar o encaminhamento de uma carta-convite aos discentes regularmente matriculados. A adesão dos programas foi voluntária, e aqueles que concordaram em participar repassaram o e-mail de recrutamento aos seus respectivos discentes.

A carta-convite apresentava informações detalhadas sobre o estudo, incluindo seus objetivos, relevância e procedimentos, além de um *link* para o Termo de Consentimento Livre e Esclarecido (TCLE). Após a leitura e aceitação do TCLE, os participantes eram redirecionados para o formulário *online*, desenvolvido e disponibilizado na plataforma REDCap.

A coleta de dados foi conduzida de forma totalmente virtual, por meio da plataforma REDCap, a qual assegura altos padrões de segurança, acessibilidade e eficiência no armazenamento e processamento das informações. O uso dessa plataforma garantiu o anonimato dos participantes e a integridade dos dados durante todas as etapas da pesquisa, promovendo confiabilidade e proteção das informações coletadas. As informações foram armazenadas de forma criptografada, com autenticação por senha e acesso restrito à equipe responsável, para garantir a confidencialidade dos dados conforme os princípios éticos aplicáveis às pesquisas com seres humanos.

### Tratamento e análise dos dados

Os dados foram compilados e exportados para uma planilha eletrônica, por meio do programa *Excel*® *Windows*. Houve dupla-digitação independente e verificação dos erros e inconsistências. As análises estatísticas foram realizadas pelo *software* R versão 4.2.3, com o suporte do pacote *Vector Generalized Additive Models* (VGAM).

Conduziu-se a análise em duas etapas principais. Na primeira, realizou-se uma análise bivariada para identificar associações estatisticamente significativas (α = 0,05) entre as variáveis independentes e os desfechos (prorrogação de prazo e trancamento de matrícula). O Teste da Razão de Verossimilhanças foi utilizado para avaliar a significância de cada variável nas etapas de inclusão e exclusão do modelo, permitindo comparações entre os modelos ajustados.

Na etapa subsequente, utilizou-se o modelo de regressão logística binária para identificar os fatores associados aos desfechos investigados. Esse modelo foi escolhido por sua adequação na análise de variáveis categóricas e desfechos binários, permitindo a estimativa de razões de chances (*Odds Ratios*) e seus intervalos de confiança (IC95%). O nível de significância adotado para as análises foi de 5% (α = 0,05).

Para a composição dos modelos finais de regressão, foram incluídas apenas as variáveis que apresentaram associação estatisticamente significativa na análise bivariada. Em seguida, aplicou-se o teste da razão de verossimilhanças para avaliar a permanência das variáveis nos modelos ajustados. Essa estratégia visou garantir parcimônia e robustez estatística, assim evitando sobreajuste. Embora diversas condições clínicas e acadêmicas tenham sido inicialmente consideradas, apenas aquelas que se mantiveram estatisticamente significativas foram incluídas nas análises multivariadas.

Neste estudo, todos os fatores estatisticamente associados aos desfechos (prorrogação e trancamento) foram denominados fatores associados. Para fins de interpretação, aqueles com razão de chance (OR) > 1 foram considerados fatores de risco, e aqueles com OR < 1, fatores de proteção.

### Aspectos éticos

O estudo foi apreciado e aprovado pelo Comitê de Ética em Pesquisa, sob o Certificado de Apresentação para Apreciação Ética (CAAE) nº: 56048822.9.0000.5393 e parecer nº 5.384.965. Foram respeitados todos os preceitos estabelecidos na Resolução 466/12 do Conselho Nacional de Saúde.

## Resultados

Da amostra de 5.245 estudantes dos quais foram obtidas respostas válidas relacionadas à prorrogação de prazo, identificou-se prevalência de 38,7% na solicitação de prorrogação durante a pandemia de COVID-19. A prorrogação foi, predominantemente, solicitada por estudantes que tinham entre 18 e 39 anos (38%), eram solteiros (38,8%), não tinham filhos (39%), declararam não ter renda (46,2%) nem afiliação religiosa (42,2%), identificavam-se como pessoas não binárias (40,5%), não heterossexuais (42,5%), identificados como “outras” categorias raciais (46,7%) e que viviam com duas a três pessoas (39,6%).

No contexto acadêmico, a prorrogação obteve maior prevalência entre doutorandos (44,9%), especialmente na Região Sudeste (39,2%), nas áreas de Ciências Biológicas (49,3%) e Engenharias (46,6%), bolsistas (39,7%) e aqueles com emprego não vinculado (41,3%). Além disso, esses pós-graduandos não precisavam viajar para estudar (39,7%), dedicavam mais de 40 horas aos estudos (42,0%), não tiveram aulas remotas (60,1%), consideraram aulas remotas inferiores às aulas presenciais (40,1%), sentiram que sua formação foi prejudicada (45,2%) e expressaram insatisfação com o ensino *on-line* (48,2%).

No contexto de saúde relacionado à COVID-19 dos discentes, verificou-se maior prevalência de prorrogações entre aqueles que manifestaram medo de contrair e/ou transmitir COVID-19 (38,4%) e faziam parte do grupo de risco incluindo tabagismo anterior à pandemia (45,7%), gestantes (48,2%), com hipertensão arterial (23,2%) e problemas renais (50,9%).

As prorrogações de prazos também foram mais frequentes entre aqueles que fizeram uso de medicamentos em geral, sem prescrição médica (40,9%), utilizaram medicamentos para se proteger da COVID-19 por conta própria (37,9%), autorreferiram testes positivos para COVID-19 (39,2%), residiam com pessoas que testaram positivo para COVID-19 e que desenvolveram sintomas graves e internação (47,8%) e foram ao óbito (47,7%).

Ademais, no grupo de participantes que realizaram prorrogação de prazos, muitos relataram ter utilizado os serviços de saúde três ou mais vezes no último ano (39,7%). Além disso, grande parte recebeu acompanhamento médico durante a pandemia (41,2%) e muitos referiram problemas de saúde mental (45,3%).

Em relação à saúde mental, houve elevada prevalência de prorrogação do prazo entre aqueles que disseram não ter se sentido bem psicologicamente no último ano (38,8%); passaram por acompanhamento psicológico antes da pandemia (40,2%), sobretudo por Transtorno Afetivo Bipolar (TAB) (50,9%), Transtorno de Ansiedade Generalizada (TAG) (41,6%) e depressão (38,3%).

Observou-se ainda elevada prevalência entre participantes que, durante a pandemia, fizeram acompanhamento para os transtornos mentais (44,3%), sendo os mais recorrentes o Transtorno de Estresse Pós-Traumático (TEPT) (51,6%), TAB (51,1%) e Síndrome do Pânico (SP) (50,2%), bem como naqueles que tinham algum membro da família com transtorno mental (39,9%), sendo mais frequente no cônjuge (43,7%). Além disso, a prorrogação foi observada naqueles com diagnóstico de transtorno mental (44,4%), diagnósticos de transtorno de TEPT (49,5%), TAB (48,4%) e SP (46,1%), naqueles que relataram não se sentir bem psicologicamente durante a pandemia e precisaram se afastar (49,9%), nos discentes com TMC positivo (42,9%) e naqueles que fizeram uso de psicofármacos durante a pandemia (50,8%), sendo os mais frequentes os potencializadores de cognição (56,2%), antidepressivos (45,2%) e ansiolíticos (43,3%).

O modelo final da análise de regressão logística revelou os fatores de risco e de proteção dos pós-graduandos que realizaram prorrogação de prazo para conclusão de curso de pós-graduação *stricto sensu* (Tabela 1).

**Tabela 1-:** Análise de regressão logística final dos fatores associados à prorrogação de prazo para conclusão de curso de pós-graduação *stricto sensu* (N = 5.245). Ribeirão Preto, SP, Brasil, 2022

**Variáveis**	b*	**SE-** b ^†^	**Z-value** ^‡^	p ^§^	**OR** ^||^	**IC** **(95%)** ^¶^	
*(Intercept)*	-0,95	0,31	-3,03	0,0025			
Região Nordeste	-0,22	0,13	-1,71	0,09	0,80	0,62	1,03
Região Norte	-0,24	0,21	-1,18	0,23	0,78	0,52	1,17
Região Sudeste	0,02	0,10	0,20	0,84	1,02	0,83	1,26
Região Sul	-0,04	0,14	-0,30	0,76	0,96	0,72	1,27
Doutorado	0,38	0,07	5,45	<0,0010	1,47	1,28	1,69
Curso Engenharia	0,40	0,14	2,97	0,0030	1,50	1,15	1,96
Curso de Biológicas	0,52	0,11	4,52	<0,0010	1,67	1,34	2,09
Ter aulas remotas	-0,94	0,29	-3,24	0,0012	0,39	0,22	0,68
Percepção de prejuízos na formação	0,76	0,08	9,39	<0,0010	2,14	1,83	2,51
Grupo de risco para COVID - hipertensos	-0,96	0,35	-2,72	0,0066	0,38	0,18	0,74
Necessidade de acompanhamento psicológico	0,35	0,07	4,74	<0,0010	1,41	1,22	1,63
Necessidade de afastamento das atividades	0,51	0,08	6,78	<0,0010	1,68	1,44	1,95
Uso de fármacos potencializadores de cognição	0,63	0,21	2,96	0,0031	1,88	1,24	2,87
Positivo para transtorno mental comum	0,27	0,08	3,41	˂0,0016	1,31	1,12	1,54

*b = Coeficiente de regressão não padronizado; ^†^SE-b = Erro padronizado; ^‡^Z-value = Coeficiente de regressão padronizado; ^§^p = Probabilidade associada ao teste estatístico; ^||^OR = *Odds Ratio* - Razão de Chances; ^¶^IC = Intervalo de confiança

Para a análise do trancamento de curso, foram consideradas 5.286 respostas válidas. Deste total, 254 participantes (4,8%) relataram ter realizado o trancamento da matrícula durante a pandemia, constituindo os casos positivos do desfecho. O maior percentual foi observado entre alunos com 60 anos ou mais (11,9%), que tinham companheiros (a) (5,7%), com um filho ou menos (8,8%), que se autodeclararam racialmente como “outros” (8,5%).

O trancamento foi mais elevado entre pós-graduandos nas Regiões Norte (6,7%), Centro-Oeste (6,0%) e Sul (4,9%). Dentro da grande área de conhecimento CAPES de Engenharias (7,5%), predominaram aqueles não bolsistas (6,1%), que tinham emprego formal (6,5%), dedicavam menos de 40 horas semanais aos estudos (4,9%), não tiveram aula remota (8,0%), relataram prejuízos em sua formação (5,7%) e insatisfação com o ensino *on-line* (6,0%).

Para o trancamento de curso, a prevalência foi maior entre pós-graduandos pertencentes ao grupo de risco da COVID-19 (8,0%), principalmente entre aqueles com problemas renais (18,9%) e gestantes (12,3%). Também foi mais elevado em indivíduos que necessitaram de internação por COVID-19 (15,2%), conviviam com pessoas que testaram positivo para a doença (5,4%), apresentaram sintomas graves que exigiram hospitalização (9,8%) e buscaram serviços de saúde três ou mais vezes no último ano (5,4%).

O trancamento também foi mais frequente entre os que realizaram acompanhamento médico durante a pandemia (6,4%), sobretudo nos grupos com problemas vasculares (10,1%), gastrointestinais (7,8%) e de saúde mental (7,7%).

Ainda no contexto de saúde mental, prevalências mais elevadas para o trancamento foram observadas entre os discentes que relataram não se sentir bem psicologicamente no último ano (5,0%), que procuraram atendimento psicológico antes da pandemia com relatos de diagnósticos de TAB (17,0%) e depressão (7,2%) e que fizeram acompanhamento psicológico antes da pandemia (5,5%) e já acompanhavam para tratar esquizofrenia anteriormente à pandemia (33,3%).

Ademais, o trancamento também foi mais observado entre os que fizeram acompanhamento psicológico durante a pandemia (6,6%), com diagnósticos de TEPT (17,4%), TAB (17,0%) e SP (10,5%), que tinham um familiar com diagnóstico de transtorno mental (5,8%), com destaque para filhos (14,1%) e cônjuges (8,6%); naqueles que já tinham diagnósticos de transtorno mental (8,5%), com destaque para TEPT (16,2%), TAB (12,9%) e depressão (9,9%); nos que precisaram se afastar de suas atividades por não se sentirem bem (8,7%) e naqueles com TMC positivo (5,9%), entre aqueles que utilizavam psicofármacos antes da pandemia (7,5%) e os que passaram a utilizá-los durante esse período (10,6%). Como exemplos de psicofármacos mais utilizados, emergiram os sedativos (11,5%), os antipsicóticos (10,7%) e os potencializadores de cognição (10,2%).

O modelo final da análise de regressão logística revelou os fatores de risco e de proteção dos pós-graduandos que solicitaram o trancamento de matrícula do curso de pós-graduação *stricto sensu* (Tabela 2).

**Tabela 2-:** Modelo de regressão logística final dos fatores associados ao trancamento de curso de pós-graduação *stricto sensu* (N = 5.286). Ribeirão Preto, SP, Brasil, 2022

**Variáveis**	b*	**SE-** b ^†^	**Z-value** ^‡^	p ^§^	**OR** ^||^	**IC** **(95%)** ^¶^	
*(Intercept)*	=4,99	0,32	-15,67	p<0,0010			
Região Nordeste	-0,11	0,28	-0,40	0,69	0,90	0,52	1,54
Região Norte	0,69	0,36	1,93	0,05	2,00	0,96	3,97
Região Sudeste	0,17	0,22	0,78	0,44	1,19	0,78	1,84
Região Sul	-0,03	0,31	-0,08	0,94	0,98	0,52	1,78
Quantidade de filhos/um	1,03	0,19	5,33	p<0,0010	2,81	1,91	4,08
Quantidade de filhos/dois ou mais	0,60	0,24	2,49	0,013	1,83	1,12	2,90
Engenharia	0,75	0,24	3,14	0,0017	2,11	1,30	3,31
Ser bolsista	-0,69	0,16	-4,23	p<0,0010	0,50	0,36	0,69
Percepção de prejuízos na formação	0,65	0,20	3,26	0,0011	1,91	1,31	2,85
Diagnóstico autorrelatado de transtorno mental	0,88	0,16	5,40	p<0,0010	2,41	1,76	3,33
Necessidade de afastamento das atividades	1,24	0,18	6,95	p<0,0010	3,44	2,44	4,91
Positivo para TMC**	0,31	0,21	1,50	0,13	1,36	0,92	2,07

*b = Coeficiente de regressão não padronizado; ^†^SE-b = Erro padronizado; ^‡^Z-value = Coeficiente de regressão padronizado; ^§^p =Probabilidade associada ao teste estatístico; ^||^OR = *Odds Ratio* - Razão de Chances; ^¶^IC = Intervalo de confiança; ^**^TMC = Transtorno Mental Comum

## Discussão

Os resultados deste estudo indicam que a prorrogação de prazo e o trancamento de matrícula na pós-graduação *stricto sensu* no Brasil estiveram diretamente associados aos desafios acadêmicos, financeiros e de saúde mental no período investigado. A pandemia da COVID-19 agravou esses fatores, ao expor as vulnerabilidades estruturais no ensino superior^(20)^ e dificultar a continuidade dos estudos em diferentes áreas do conhecimento^(6-7,11-12,21-23)^. A crise sanitária impôs transformações abruptas nas instituições acadêmicas, o que exigiu adaptações nos modelos de ensino e no suporte aos discentes.

Entre os principais fatores de risco relacionados à prorrogação de prazo, destacam-se estar matriculado no doutorado, cursar Engenharias ou Ciências Biológicas, percepção de prejuízos na formação e apresentar problemas de saúde mental. O trancamento de matrícula, por sua vez, esteve relacionado a estar cursando pós-graduação em Engenharia, à percepção de prejuízos na formação intensificados pela pandemia, ao diagnóstico de transtornos mentais e à necessidade de afastamento acadêmico.

Assim, enquanto a prorrogação de prazo reflete uma tentativa de continuidade acadêmica, mesmo diante de adversidades, o trancamento representa uma interrupção mais significativa do percurso formativo, geralmente associada às condições mais graves de comprometimento emocional, institucional ou de saúde. Embora ambos os desfechos estejam vinculados à vulnerabilidade estudantil, eles demandam respostas institucionais distintas, ajustadas ao grau de comprometimento e às características do contexto vivenciado pelos discentes.

A rápida transição para o ensino remoto revelou dificuldades estruturais importantes, como a falta de infraestrutura tecnológica e a exclusão digital de estudantes com menor poder aquisitivo^(3,7,14)^. Tais obstáculos afetaram de forma desigual os programas de pós-graduação, sendo particularmente evidentes em áreas que dependem de atividades práticas, como as Engenharias, nas quais a migração para o ensino remoto limitou significativamente o desenvolvimento de pesquisas experimentais, sobretudo aquelas que exigiam acesso aos laboratórios e às atividades de campo^(24)^.

Mesmo antes da pandemia, estudantes de doutorado já enfrentavam desafios para manter o bem-estar físico e mental diante da intensa carga acadêmica e da falta de tempo para autocuidado^(9,15,25)^. Com a pandemia, essas dificuldades agravaram-se pela necessidade de conciliar ensino remoto, continuidade das pesquisas e responsabilidades familiares, muitas vezes sem suporte financeiro ou social adequado^(11-12,26-27)^.

A sobrecarga acadêmica, as incertezas acerca do futuro e as perdas pessoais intensificaram transtornos como ansiedade e depressão, destacando a necessidade de um suporte institucional mais efetivo^(5,15,28)^. A elevada exigência acadêmica e a pressão por produtividade afetaram significativamente a saúde mental dos pós-graduandos, que passaram a apresentar altos níveis de estresse com consequente comprometimento da permanência e desempenho acadêmico^(5,11)^. Ademais, a dificuldade de equilibrar as demandas acadêmicas, pessoais e financeiras pode ser reconhecida como um obstáculo crítico para o bem-estar desses estudantes e reforçar a importância de estratégias institucionais para a mitigação desses impactos^(6,11-12)^.

Destaca-se que a pandemia agravou significativamente o cenário de sofrimento psíquico entre estudantes, especialmente daqueles em situação de vulnerabilidade socioeconômica, em razão do isolamento social e da redução das interações presenciais^(3-4,7,24)^. A prevalência de transtornos mentais comuns (TMC) entre pós-graduandos brasileiros alcançou 63,5%, associada aos fatores como dificuldades acadêmicas, insegurança financeira, prorrogação de prazos e afastamentos temporários dos estudos^(5,27)^. Em resposta às pressões vivenciadas, muitos recorreram ao uso de psicofármacos sem acompanhamento médico, o que evidencia a urgência da implementação de políticas institucionais que fortaleçam o suporte em saúde mental e incentivem estratégias de enfrentamento mais seguras e eficazes^(9,15,25)^.

Estudos indicam que, durante a pandemia de COVID-19, houve um aumento expressivo nos sintomas de ansiedade e depressão entre estudantes universitários, especialmente entre aqueles vinculados à área da saúde. A prevalência de sintomas depressivos variou entre 31% e 38%, enquanto os de ansiedade alcançaram cerca de 41%^(29)^. Entre pesquisadores de pós-graduação, aproximadamente 70% relataram sofrimento psicológico de leve a grave, apesar dos níveis mais elevados de letramento em saúde mental^(30)^. Esses achados ressaltam a importância de políticas institucionais para garantir atenção contínua à saúde mental desse público, particularmente diante da exposição prolongada aos múltiplos estressores acadêmicos e pessoais^(25,27,29-30)^.

A presença de dificuldades acadêmicas intensificadas pela pandemia, a migração para o ensino remoto e a sobrecarga vivenciada por docentes e discentes durante esse período pode ter contribuído para maior vulnerabilidade emocional dos discentes, evidenciando a necessidade de estratégias institucionais para fortalecer o suporte acadêmico e a saúde mental^(11,24)^. Segundo pesquisas, uma relação positiva entre orientador e pós-graduando pode reduzir significativamente o estresse acadêmico (β = -0.27, p < 0.01), sendo esse efeito parcialmente mediado pela autoeficácia (β = -0.14, p < 0.01) e moderado pela resiliência psicológica (β = -0.10, p < 0.01)^(26)^. Nesse contexto, a qualidade da orientação acadêmica e a existência de um ambiente de suporte mostram-se fundamentais para minimizar os impactos negativos da pós-graduação^(11,26)^ e reduzir a necessidade de prorrogações e trancamentos^(9,12)^.

Entre os participantes que relataram acesso às aulas remotas, observou-se menor frequência de prorrogação do curso, o que justifica o resultado deste atuar como fator protetor nesse subgrupo, apesar de representar somente 40% da amostra. A despeito das dificuldades para adaptação ao ensino remoto, a participação em aulas virtuais mostrou-se um fator protetor contra a prorrogação de prazos, possivelmente pela flexibilidade proporcionada por esse modelo. A possibilidade de gerenciar melhor o tempo, acessar novas oportunidades de aprendizado e aumentar a produtividade favoreceu a continuidade acadêmica dos estudantes^(6,21-22,24)^.

Estudos prévios já apontavam que a eliminação da necessidade de deslocamento físico facilitou a permanência de muitos pós-graduandos em seus cursos, ao reduzir barreiras logísticas e otimizar o tempo dedicado às atividades acadêmicas^(4,24)^. Além disso, a ampliação do acesso aos cursos e conteúdos antes restritos aos determinados locais, juntamente com a flexibilização dos horários de estudo, beneficiou especialmente os estudantes que conciliavam atividades acadêmicas e profissionais, tornando o ensino mais acessível e adaptável às suas realidades^(11,24,26)^.

Um achado merecedor de destaque é o fato de que o diagnóstico prévio de hipertensão arterial sistêmica, embora classificado como fator de risco para complicações da COVID-19, foi identificado neste estudo como um fator protetor contra a prorrogação de curso na pós-graduação *stricto sensu*. Uma possível explicação para esse resultado é que estudantes com hipertensão, por estarem em acompanhamento médico contínuo, tendem a apresentar maior adesão **às** práticas de autocuidado, maior consciência sobre a gestão do estresse e melhor estruturação de rotinas pessoais e acadêmicas, mesmo diante das adversidades impostas pela pandemia.

Embora a hipertensão seja geralmente associada aos piores desfechos clínicos^(31)^, ela pode também funcionar como um marcador de maior vigilância à saúde, particularmente em indivíduos com acesso a serviços médicos e suporte terapêutico^(32)^. De acordo com estudos recentes, fatores como atividade física, resiliência psicológica e estratégias de enfrentamento positivas podem mitigar os efeitos do estresse sobre a saúde mental e acadêmica de pessoas hipertensas^(31-32)^. Assim, o diagnóstico prévio pode refletir não apenas uma condição crônica, mas também uma trajetória de adaptação e cuidado, o que pode ter favorecido a continuidade das atividades acadêmicas durante o período pandêmico.

Ainda assim, trata-se de um achado não usual e pouco explorado na literatura científica. Faz-se, portanto, necessária investigação complementar em estudos futuros para elucidar os mecanismos envolvidos e avaliar se esse padrão confirma-se em outras populações acadêmicas ou contextos institucionais.

As implicações deste estudo reforçam a necessidade urgente de políticas institucionais voltadas à promoção da saúde mental e à mitigação dos impactos da sobrecarga acadêmica na pós-graduação. Estratégias eficazes incluem a ampliação do suporte psicológico, a capacitação de orientadores, a flexibilização acadêmica e o fortalecimento da assistência estudantil, no sentido de garantir condições mais equitativas para a permanência dos discentes^(5,8-9)^. Ademais, destaca-se a importância da reformulação das matrizes curriculares, para torná-las mais eficazes e flexíveis, de modo a assegurar a sustentabilidade da pós-graduação e reduzir a incidência de prorrogações e evasões^(21)^.

O fortalecimento do suporte psicológico revelou-se essencial, envolvendo a ampliação dos serviços de atendimento nas universidades, a criação de grupos de apoio, ações de acolhimento e programas de mentoria acadêmica. Essas iniciativas poderão contribuir para um ambiente universitário mais saudável e reduzir os impactos negativos da pressão acadêmica sobre os estudantes^(21,25)^.

Embora a relação entre orientador e estudante não tenha sido diretamente avaliada neste estudo, a literatura destaca sua importância como fator determinante para o sucesso acadêmico^(26-27)^. A capacitação de orientadores revela-se, portanto, fundamental. Estratégias institucionais que incentivem a formação continuada dos docentes, com foco em uma orientação mais humanizada e sensível às necessidades dos discentes, podem minimizar os impactos negativos de um acompanhamento inadequado. A implementação de diretrizes institucionais que estabeleçam boas práticas na orientação acadêmica contribui diretamente para um ambiente mais produtivo, colaborativo e menos estressante^(26-27)^.

A flexibilização acadêmica deve ser incentivada, a fim de permitir ajustes nos prazos de conclusão de disciplinas, dissertações e teses para que os estudantes possam superar períodos de maior dificuldade sem comprometer a trajetória acadêmica. A adaptação das avaliações e a promoção de currículos mais flexíveis tornam o ensino mais acessível e sustentável, com consequente redução das taxas de prorrogação de prazos e trancamento de matrículas^(28)^.

O apoio financeiro e a assistência estudantil são fundamentais para garantir a permanência dos pós-graduandos. A ampliação de bolsas de pesquisa, auxílios financeiros para transporte e moradia e a criação de programas de financiamento estudantil podem minimizar a evasão acadêmica motivada por dificuldades econômicas. A segurança financeira, comprovadamente, reduz a vulnerabilidade dos estudantes e melhora a capacidade de enfrentar os desafios da pós-graduação^(5,21)^.

Outrossim, a promoção do bem-estar e de estratégias de enfrentamento saudáveis deve ser incentivada por meio de atividades culturais, esportivas e sociais, que atuam como fatores de proteção à saúde mental. Programas de mentoria entre pares, incentivo à prática de atividades físicas e a criação de espaços de convivência dentro das universidades auxiliam na redução do estresse e no fortalecimento das redes de apoio social^(26,28)^.

Por fim, o monitoramento contínuo da saúde mental dos discentes deve ser implementado por meio de pesquisas institucionais periódicas, para subsidiar a elaboração de políticas eficazes de suporte. O desenvolvimento de plataformas anônimas para denúncia de más práticas acadêmicas e assédio pode contribuir para um ambiente acadêmico mais seguro e acolhedor. A adoção de estratégias institucionais que priorizem tanto a excelência acadêmica quanto o bem-estar dos discentes é essencial para um sistema educacional mais sustentável e equitativo^(3,7,9,11,25,29-30)^.

Mesmo com as contribuições deste estudo, algumas limitações devem ser consideradas. O delineamento transversal impede a determinação de causalidade entre os fatores analisados e os desfechos acadêmicos, limitando-se à identificação de associações. A coleta de dados autodeclarados pode ter gerado viés de resposta, especialmente em questões sensíveis, como saúde mental e insegurança financeira. Além disso, a ausência de dados qualitativos restringe uma compreensão mais aprofundada sobre as percepções dos pós-graduandos em relação ao suporte institucional e às estratégias individuais de enfrentamento.

A representatividade da amostra pode não abranger todas as realidades da pós-graduação brasileira, o que indica a necessidade de estudos futuros para contemplar diferentes contextos institucionais e regionais. Pesquisas longitudinais são essenciais para avaliar o impacto de políticas institucionais na permanência acadêmica e na saúde mental dos discentes ao longo do tempo. Além disso, abordagens qualitativas podem aprofundar a análise dos desafios enfrentados e das estratégias utilizadas pelos estudantes para lidar com as adversidades acadêmicas^(25)^.

Diante dos desafios identificados, é essencial que as instituições de ensino invistam em estratégias integradas promotoras de saúde mental e fortalecedoras da permanência dos pós-graduandos. A ampliação do suporte psicológico, a capacitação humanizada de orientadores, a flexibilização acadêmica, o investimento em assistência estudantil e a promoção do bem-estar devem compor um conjunto de ações contínuas e acessíveis, ajustadas à diversidade sociocultural e digital dos estudantes. Tais medidas não apenas reduzem a evasão e o sofrimento psíquico como fortalecem o sistema educacional, contribuindo para um percurso acadêmico mais equitativo, saudável e favorável ao desenvolvimento científico e profissional.

## Conclusão

Este estudo evidenciou que a prorrogação do prazo e o trancamento de curso na pós-graduação *stricto sensu*, durante a pandemia de COVID-19, foram influenciados por fatores de risco e de proteção de natureza acadêmica, pessoal e psicossocial. A prorrogação esteve associada, principalmente, ao vínculo com programas de doutorado, às áreas de Engenharias e Ciências Biológicas, à percepção de prejuízos na formação e à presença de transtornos mentais. Em contrapartida, a participação em aulas remotas e o diagnóstico prévio de hipertensão atuaram como fatores de proteção, embora limitações como a precariedade da infraestrutura tecnológica e a redução da interação social tenham sido desafios relevantes para muitos discentes.

O trancamento esteve relacionado aos fatores como parentalidade, dificuldades vivenciadas durante a pandemia, histórico de afastamento e problemas de saúde mental, enquanto o recebimento de bolsa emergiu como fator protetivo. Os resultados destacam a centralidade da saúde mental na permanência acadêmica e reforçam a necessidade de políticas institucionais voltadas ao apoio psicossocial, à redução das desigualdades socioeconômicas, à flexibilização de trajetórias formativas e à promoção de ambientes acadêmicos mais inclusivos, sobretudo em contextos de crise.

## Data Availability

O conjunto de dados que dá suporte aos resultados deste estudo não está disponível publicamente.

# Translation: Factores asociados a las prórrogas y a las suspensiones de la postgrado *stricto sensu* en Brasil durante la pandemia de COVID-19: énfasis en la salud mental

**DOI:** 10.1590/1518-8345.7988.4752

**Published:** 2026

**Authors:** Letuza Ribeiro Teixeira, Adriana Inocenti Miasso, Marina Aleixo Diniz Rezende, Nayara Paula Fernandes Martins Molina, Vanessa da Silva Carvalho Vila

**Affiliations:** 1 Pontifícia Universidade Católica de Goiás, Goiânia, GO, Brasil. Pontifícia Universidade Católica de Goiás GO Goiânia Brasil; 2 Universidade de São Paulo, Escola de Enfermagem de Ribeirão Preto, Centro Colaborador de la OPS/OMS para el Desarrollo de la Investigación en Enfermería, Ribeirão Preto, SP, Brasil. Universidade de São Paulo Escola de Enfermagem de Ribeirão Preto Centro Colaborador de la OPS/OMS para el Desarrollo de la Investigación en Enfermería SP Ribeirão Preto Brasil; 3 Becaria del Conselho Nacional de Desenvolvimento Científico e Tecnológico (CNPq), Brasil Becaria del Conselho Nacional de Desenvolvimento Científico e Tecnológico Brasil; 4 Becaria de la Coordenação de Aperfeiçoamento de Pessoal de Nível Superior (CAPES), Brasil. Becaria de la Coordenação de Aperfeiçoamento de Pessoal de Nível Superior Brasil

**Keywords:** Educación de Posgrado, Salud Mental, COVID-19, Universidades, Enseñanza, Factores de Riesgo

## Abstract

analizar los factores asociados a la prórroga del plazo de finalización y a la suspensión de cursos entre estudiantes brasileños matriculados en programas de postgrado *stricto sensu* durante la pandemia de COVID-19, con énfasis en la salud mental.

estudio transversal analítico realizado con 5.286 estudiantes de postgrado matriculados en 2022. Los datos se recopilaron mediante un formulario electrónico con información sociodemográfica, académica y de salud, que incluía antecedentes de trastornos mentales. Se realizó un análisis estadístico descriptivo e inferencial, con regresión logística utilizando *el software* R.

se constató que el 38% de los participantes extendió sus cursos y el 4,8% suspendió su matrícula. Las extensiones se asociaron con la realización de carreras de Doctorado, Ingeniería o Ciencias Biológicas, el reporte de dificultades en la formación y la presentación de problemas de salud mental. La participación en clases remotas y un diagnóstico previo de hipertensión emergieron como factores protectores. La suspensión de estudios se asoció con tener hijos, estudiar Ingeniería, enfrentar dificultades durante la pandemia, presentar problemas de salud mental y antecedentes de abandono académico. Ser becario fue un factor protector y redujo las probabilidades de suspensión en un 50%

los hallazgos indican que las prórrogas y suspensiones están relacionadas con factores académicos, personales y de salud y resaltan la necesidad de políticas institucionales que promuevan el apoyo integral, con especial atención a la salud mental y la retención académica de los estudios de postgrado.

## Destacados

**(1)** El 38% de los estudiantes de postgrado prorrogaron el curso y el 4,8% suspendieron sus matrículas. **(2)** Dificultades académicas y de salud mental impactaron en las prórrogas y suspensiones. **(3)** Ser becario redujo en un 50% la posibilidad de suspender los estudios de postgrado. **(4)** Las clases remotas fueron un factor protector para las prórrogas de los cursos. **(5)** Las políticas educativas deben promover el apoyo académico y el bienestar mental.

## Introducción

A lo largo de sus 50 años de existencia, el Sistema Nacional de Postgrado de Brasil se ha consolidado como referente en formación avanzada en América Latina, desempeñando un papel esencial en la formación de académicos y profesionales para el avance científico, tecnológico e innovador del país^(1-4)^. Sin embargo, la pandemia de COVID-19 ha impuesto importantes desafíos a los estudiantes de postgrado *stricto sensu*, al profundizar las desigualdades estructurales y afectar la salud mental^(5-9)^.

Un estudio reciente reveló que el 63,5 % de los estudiantes de postgrado brasileños presentaron trastornos mentales comunes (TMC) durante la pandemia, especialmente mujeres, jóvenes y estudiantes de maestría, en particular aquellos que enfrentaron dificultades financieras y retrasos académicos^(5)^. A nivel mundial, los investigadores experimentaron un mayor estrés y ansiedad debido a la inseguridad financiera, la pérdida de oportunidades laborales y el aislamiento social, lo que reforzó la necesidad de apoyo institucional^(6,10-12)^.

En Brasil, el acceso restringido a los laboratorios, los retrasos en los cronogramas de investigación y la transición al aprendizaje remoto de emergencia se han convertido en obstáculos críticos para la continuidad de la formación académica y han incrementado la sensación de sobrecarga y soledad entre los estudiantes^(5,13-14)^. La presión por la productividad en un escenario de incertidumbre ha intensificado el malestar psicológico, que a menudo requiere el retiro temporal de las actividades académicas^(6,11)^.

A este escenario se sumaron los impactos de las dificultades financieras, que se agravaron durante la pandemia y se asociaron con un aumento en los síntomas depresivos, lo que demuestra aún más la importancia de la estabilidad económica para mantener la salud mental de los estudiantes y la necesidad de intervenciones específicas para apoyar a las personas vulnerables durante las crisis^(3-7)^. Las investigaciones también han identificado que los estudiantes en programas de postgrado *stricto sensu* enfrentaron repercusiones significativas en sus vidas personales y académicas, particularmente debido a la necesidad de adaptarse al aprendizaje remoto de emergencia y la necesidad de abordar problemas sociales, económicos y psicológicos complejos^(11,15)^.

Ante esta situación, muchos han recurrido a solicitar prórrogas o extensiones de los plazos y suspender su matrícula. En Brasil, estos instrumentos están regulados por las instituciones de educación superior y siguen las normas establecidas por la *Coordenação de Aperfeiçoamento de Pessoal de Educação Superior* (CAPES). Prorrogar el plazo de finalización permite a los estudiantes aumentar el tiempo máximo estipulado para completar un curso, mientras que suspender su matrícula implica suspender temporalmente sus actividades académicas, manteniendo su vínculo con la institución.

Ambos instrumentos son esenciales para garantizar la continuidad de la formación académica y brindar el apoyo necesario a los estudiantes para superar las adversidades sin comprometer el rigor ni la calidad de la enseñanza. En el contexto de la pandemia, se reiteró la relevancia de estas estrategias, debido al reconocimiento y la respuesta a la complejidad de los desafíos que se impusieron abruptamente en las trayectorias de los estudiantes^(15)^.

En este contexto, este estudio tuvo como objetivo analizar los factores asociados con la extensión de los plazos de finalización y a la suspensión de cursos entre estudiantes brasileños matriculados en programas de postgrado *stricto sensu* durante la pandemia de COVID-19, con énfasis en la salud mental. Este análisis busca fundamentar políticas y estrategias que garanticen la salud mental y la retención académica de los discentes, especialmente en tiempos de crisis.

## Método

### Tipo y lugar de estudio

Estudio transversal analítico, de acuerdo con las directrices de *Strengthening the Reporting of Observational Studies in Epidemiology* (STROBE)^(16)^. La investigación se realizó virtualmente y abarcó todo el territorio brasileño, utilizando la plataforma *de Research Electronic Data Capture* (REDCap). La recopilación de datos tuvo lugar entre mayo y julio de 2022.

### Población y muestra

La población del estudio se conformó por discentes de programas de postgrado *stricto sensu* en Brasil en 2022. Según datos de la *Coordenação de Aperfeiçoamento de Pessoal de Educação Superior* (CAPES), en 2019, Brasil contaba con 388.629 estudiantes distribuidos en 4.565 programas de postgrado en 498 instituciones de educación superior (IES), lo que incluía maestrías y doctorados académicos y profesionales^(17)^.

El cálculo del tamaño de la muestra, considerando todo el territorio nacional, se basó en una prevalencia estimada del 50%, una precisión del 1,5% y un intervalo de confianza del 95%, lo que resultó en una muestra mínima requerida de 4222 participantes. Cabe destacar que, en ausencia de información específica sobre la prevalencia de los resultados, como es el caso de los estudiantes de postgrado, se recomienda el cálculo con una prevalencia del 50%, ya que garantiza que el tamaño de la muestra sea suficiente para cualquier valor de prevalencia observado^(18)^.

Fueron incluidos en el estudio estudiantes inscriptos en programas de postgrado académicos y profesionales *stricto sensu,* mayores de 18 años, de ambos sexos, residentes en Brasil y con acceso a internet. Se excluyó a quienes no completaron completamente el formulario de recolección de datos. El estudio arrojó un total de 5.334 respuestas. Para el análisis de las extensiones de plazo, se consideraron válidas 5.245 respuestas a la pregunta específica para este resultado. Para el análisis de la suspensión de la matrícula, se incluyeron 5.286 respuestas válidas a la pregunta respectiva. Los dos resultados se analizaron de forma independiente, y los participantes que informaron haber experimentado ambos eventos se incluyeron en ambos análisis, según sus respuestas individuales a cada variable.

### Variables de estudio

Las variables dependientes analizadas fueron la ocurrencia de prórrogas de plazos y la suspensión de matrículas. Se definió la prórroga de plazo como la solicitud formal para extender el período originalmente estipulado para la finalización del curso, mientras que la suspensión de matrícula se caracterizó por la suspensión temporal y formal de las actividades académicas por parte del estudiante.

Las variables independientes se organizaron en tres categorías principales: sociodemográficas, académicas, antecedentes de salud y trastornos mentales. Las variables sociodemográficas incluyeron rango de edad, género, orientación sexual, estado civil, existencia y número de hijos, ingresos mensuales individuales, composición del hogar, raza/etnia y religión.

Las variables académicas abordaron el tipo de curso de postgrado *stricto sensu* (académico o profesional), la región geográfica del curso, el campo amplio del conocimiento, la condición de becario, la simultaneidad entre trabajo y estudios, la carga horaria semanal dedicada a estudios, la realización de clases a distancia, la percepción de equivalencia entre clases a distancia y presenciales, el impacto de la pandemia en la formación académica, la calidad del acceso a internet para las actividades académicas y el grado de satisfacción con la educación digital.

Las variables relacionadas con el historial de salud y los trastornos mentales abarcaron diversos aspectos, como el miedo a contraer o transmitir el coronavirus, el estado de vacunación contra el COVID-19 (incluido el número de dosis recibidas) y la pertenencia a grupos de riesgo. También se evaluó el uso de medicamentos de venta libre durante la pandemia, incluidos los de protección contra el COVID-19 y el acceso a servicios de salud durante el último año, con especial atención al seguimiento médico y psicológico.

Se investigaron aspectos específicos relacionados con el COVID-19, como la incidencia de pruebas positivas, la necesidad de hospitalizaciones, la progresión de casos positivos entre convivientes y el uso continuo de medicamentos, incluyendo psicofármacos. Además, se consideraron el historial de tabaquismo (duración del consumo y cantidad de cigarrillos al día), la automedicación, los episodios de malestar psicológico durante la pandemia, los diagnósticos previos de trastornos mentales y la necesidad de ausentarse de las actividades académicas debido a problemas psicológicos.

En el ámbito de la salud mental, se incluyeron los autoinformes de diagnósticos previos de trastornos mentales y durante la pandemia, el uso de psicofármacos, el inicio o continuación del uso de estos medicamentos durante la pandemia, su prescripción o ausencia, así como el seguimiento psicológico antes y durante la pandemia.

### Instrumentos de recolección de datos

Se utilizó un cuestionario estructurado en línea que incluía datos sociodemográficos y académicos, antecedentes de salud y trastornos mentales, desarrollado por los investigadores del proyecto original y con validación de contenido. También se utilizó el *Self-Reporting Questionnaire* (SRQ-20), propuesto por la Organización Mundial de la Salud (OMS) para el cribado de trastornos mentales comunes (TMC) y validado en Brasil para el cribado de trastornos mentales comunes en la población general^(19)^.

El SRQ-20 consta de 20 preguntas divididas en cuatro categorías: quejas somáticas, trastorno ansioso-depresivo, pérdida de energía vital y pensamientos depresivos. Las preguntas abordan síntomas físicos (cuatro preguntas) y emocionales (16 preguntas), con respuestas binarias (sí/no). Cada respuesta positiva suma un punto, generando una puntuación total de 0 a 20; las puntuaciones más altas indican una mayor probabilidad de trastornos mentales no psicóticos (TMNP). Para este estudio, se adoptaron los puntos de corte validados: ocho o más respuestas positivas para mujeres y seis o más para hombres indicaron sospecha de TMC.

### Recopilación de datos

La recopilación de datos se realizó de forma estructurada y exhaustiva. Inicialmente, se enviaron correos electrónicos a los 4592 programas de postgrado *stricto sensu* de Brasil, solicitando el envío de una carta de invitación a los estudiantes matriculados regularmente. La participación en los programas fue voluntaria, y quienes aceptaron reenviaron el correo electrónico de reclutamiento a sus respectivos estudiantes.

La carta de invitación proporcionó información detallada sobre el estudio, incluyendo sus objetivos, relevancia y procedimientos, así como un enlace al Formulario de Consentimiento Libre e Informado (FCLI). Tras leer y aceptar el FCLI, los participantes fueron redirigidos al formulario *online,* desarrollado y disponible en la plataforma REDCap.

La recopilación de datos se realizó íntegramente en línea mediante la plataforma REDCap, que garantiza altos estándares de seguridad, accesibilidad y eficiencia en el almacenamiento y procesamiento de datos. El uso de esta plataforma garantizó el anonimato de los participantes y la integridad de los datos durante todas las etapas de la investigación, promoviendo la fiabilidad y protección de la información recopilada. La información se almacenó encriptada, con autenticación mediante contraseña y acceso restringido al equipo responsable, garantizando así la confidencialidad de los datos de acuerdo con los principios éticos aplicables a la investigación con seres humanos.

### Procesamiento y análisis de datos

Los datos se recopilaron y exportaron a una hoja de cálculo con *Excel® Windows.* La carga de datos se realizó por doble entrada independiente y se verificaron los errores y las inconsistencias. Los análisis estadísticos se llevaron a cabo con el *software* R versión 4.2.3, con el apoyo del paquete *Vector Generalized Additive Models* (VGAM).

El análisis se realizó en dos etapas principales. En la primera, se realizó un análisis bivariado para identificar asociaciones estadísticamente significativas (α = 0,05) entre las variables independientes y los resultados (extensión del período y suspensión de la matrícula). Se utilizó la prueba de razón de verosimilitud para evaluar la significancia de cada variable en las etapas de inclusión y exclusión del modelo, lo que permitió realizar comparaciones entre los modelos ajustados.

En la etapa posterior, se utilizó el modelo de regresión logística binaria para identificar los factores asociados con los resultados investigados. Este modelo se seleccionó por su idoneidad para el análisis de variables categóricas y resultados binarios, permitiendo la estimación de las razones de probabilidades (*Odds Ratio*) y sus intervalos de confianza (IC del 95%). El nivel de significancia adoptado para los análisis fue del 5% (α = 0,05).

Para componer los modelos de regresión finales, solo se incluyeron las variables que mostraron una asociación estadísticamente significativa en el análisis bivariado. Posteriormente, se aplicó la prueba de razón de verosimilitud para evaluar la permanencia de las variables en los modelos ajustados. Esta estrategia buscó asegurar la parsimonia y la robustez estadística, evitando así el sobreajuste. Si bien en un principio se consideraron diversas condiciones clínicas y académicas, solo aquellas que mantuvieron la significación estadística se incluyeron en los análisis multivariados.

En este estudio, todos los factores estadísticamente asociados con los resultados (prórroga y suspensión) se denominaron factores asociados. Para fines de interpretación, aquellos con un OR > 1 se consideraron factores de riesgo y aquellos con un OR < 1, factores protectores.

### Aspectos éticos

El estudio fue evaluado y aprobado por el Comité de Ética de Investigación, con el Certificado de Presentación para Evaluación Ética (CAAE) n.º 56048822.9.0000.5393 y el dictamen n.º 5.384.965. Se respetaron todos los preceptos establecidos en la Resolución 466/12 del Consejo Nacional de Salud.

## Resultados

De la muestra de 5245 estudiantes de quienes se obtuvieron respuestas válidas sobre prórrogas, se identificó una prevalencia del 38,7% en solicitudes de prórroga durante la pandemia de COVID-19. Las prórrogas fueron solicitadas predominantemente por estudiantes de entre 18 y 39 años (38%), solteros (38,8%), sin hijos (39%), sin ingresos (46,2%) ni afiliación religiosa (42,2%), no binarios (40,5%), no heterosexuales (42,5%), pertenecientes a otras categorías raciales (46,7%) y que vivían con dos o tres personas (39,6%).

En el contexto académico, las extensiones fueron más frecuentes entre los estudiantes de doctorado (44,9%), especialmente en la Región Sudeste (39,2%), en las áreas de Ciencias Biológicas (49,3%) e Ingenierías (46,6%), becarios (39,7%) y aquellos con empleo no relacionado (41,3%). Además, los estudiantes de postgrado que no necesitaban viajar para estudiar (39,7%), dedicaban más de 40 horas a sus estudios (42,0%), no tuvieron clases remotas (60,1%), consideraron las clases remotas inferiores a las clases presenciales (40,1%), sintieron que su formación se vio perjudicada (45,2%) y expresaron insatisfacción con la enseñanza en línea (48,2%).

En el contexto de la salud relacionado con el COVID-19 entre los estudiantes, se observó una mayor prevalencia de prórrogas entre quienes manifestaron temor a contraer y/o transmitir el COVID-19 (38,4%) y formaban parte del grupo de riesgo, entre ellos tabaquismo previo a la pandemia (45,7%), mujeres embarazadas (48,2%), con hipertensión arterial (23,2%) y problemas renales (50,9%).

Las prórrogas también fueron más frecuentes entre quienes usaron medicamentos en general, sin prescripción médica (40,9%), usaron medicamentos para protegerse del COVID-19 por su cuenta (37,9%), informaron pruebas positivas para COVID-19 (39,2%), residían con personas que dieron positivo en COVID-19 y que desarrollaron síntomas graves y hospitalización (47,8%) y evolucionaron a óbito (47,7%).

Además, entre los participantes que extendieron sus plazos, muchos reportaron haber utilizado servicios de salud tres o más veces en el último año (39,7%). Asimismo, una gran proporción estuvo en seguimiento médico durante la pandemia (41,2%) y muchos reportaron problemas de salud mental (45,3%).

Respecto a la salud mental, hubo una alta prevalencia de extensión del plazo entre quienes manifestaron no haberse sentido bien psicológicamente en el último año (38,8%); haber recibido apoyo psicológico antes de la pandemia (40,2%), sobre todo por Trastorno Afectivo Bipolar (TAB) (50,9%), Trastorno de Ansiedad Generalizada (TAG) (41,6%) y depresión (38,3%).

También se observó una alta prevalencia entre los participantes que, durante la pandemia, fueron monitoreados por trastornos mentales (44,3%), siendo los más recurrentes el Trastorno de Estrés Postraumático (TEPT) (51,6%), el TAB (51,1%) y el Síndrome de Pánico (SP) (50,2%), así como en aquellos que tenían un familiar con un trastorno mental (39,9%), siendo más frecuente en el cónyuge (43,7%). Además, la extensión se observó en aquellos diagnosticados con trastornos mentales (44,4%), TEPT (49,5%), TAB (48,4%) y SP (46,1%), en aquellos que reportaron no sentirse bien psicológicamente durante la pandemia y necesitaron tomarse un tiempo (49,9%), en estudiantes con TMC positivo (42,9%) y en aquellos que usaron psicofármacos durante la pandemia (50,8%), advirtiéndose la prevalencia de los potenciadores cognitivos (56,2%), los antidepresivos (45,2%) y los ansiolíticos (43,3%).

El modelo final del análisis de regresión logística reveló los factores de riesgo y protección de los estudiantes de posgrado que solicitaron una prórroga para completar su curso de posgrado *stricto sensu* (Tabla 1).

**Tabla 1-:** Análisis de regresión logística final de los factores asociados a la prórroga del plazo para la finalización de un curso de posgrado *stricto sensu* (N = 5245). Ribeirão Preto, SP, Brasil, 2022

**Variables**	b*	**SE-** b ^†^	**Valor Z** ^‡^	p ^§^	**OR** ^||^	**CI** **(95%)** ^¶^	
*(Intercept)*	-0,95	0,31	-3,03	0,0025			
Región Nordeste	-0,22	0,13	-1,71	0,09	0,80	0,62	1,03
Región Norte	-0,24	0,21	-1,18	0,23	0,78	0,52	1,17
Región Sudeste	0,02	0,10	0,20	0,84	1,02	0,83	1,26
Región Sur	-0,04	0,14	-0,30	0,76	0,96	0,72	1,27
Doctorado	0,38	0,07	5,45	<0,0010	1,47	1,28	1,69
Carreras de Ingeniería	0,40	0,14	2,97	0,0030	1,50	1,15	1,96
Carreras de Ciencias Biológicas	0,52	0,11	4,52	<0,0010	1,67	1,34	2,09
Tener clases remotas	-0,94	0,29	-3,24	0,0012	0,39	0,22	0,68
Percepción de perjuicios en la formación	0,76	0,08	9,39	<0,0010	2,14	1,83	2,51
Grupo de riesgo de COVID: pacientes hipertensos	-0,96	0,35	-2,72	0,0066	0,38	0,18	0,74
Necesidad de apoyo psicológico	0,35	0,07	4,74	<0,0010	1,41	1,22	1.63
Necesidad de retirarse de las actividades	0,51	0,08	6,78	<0,0010	1,68	1,44	1,95
Uso de fármacos	0,63	0,21	2,96	0,0031	1,88	1,24	2.87
Positivo en trastorno mental común	0,27	0,08	3,41	˂0,0016	1,31	1,12	1.54

*b = Coeficiente de regresión no estandarizado; ^†^SE-b = Error estandarizado; ^‡^Valor Z = Coeficiente de regresión estandarizado; ^§^p = Probabilidad asociada a la prueba estadística; ^||^OR = *Odds Ratio*; ^¶^IC = Intervalo de confianza

Para el análisis de la suspensión de cursos, se consideraron 5286 respuestas válidas. De este total, 254 participantes (4,8%) informaron haber suspendido su matrícula durante la pandemia, lo que constituye los casos positivos de los resultados. El mayor porcentaje se observó entre los estudiantes de 60 años o más (11,9%), que tenían pareja (5,7%), con uno o menos hijos (8,8%) y que se autoidentificaron como racialmente “otro” (8,5%).

La tasa de suspensión fue mayor entre los estudiantes de postgrado de las regiones Norte (6,7%), Centro-Oeste (6,0%) y Sur (4,9%). Dentro del área de conocimiento de CAPES, Ingeniería (7,5%), predominaron quienes no recibieron becas (6,1%), tenían empleo formal (6,5%), dedicaban menos de 40 horas semanales a los estudios (4,9%), no tenían clases remotas (8,0%), reportaron perjuicios en su formación (5,7%) e insatisfacción con la enseñanza en línea (6,0%).

En cuanto a la suspensión de cursos, la prevalencia fue mayor entre los estudiantes de postgrado pertenecientes al grupo de riesgo de COVID-19 (8,0%), especialmente entre aquellos con problemas renales (18,9%) y mujeres embarazadas (12,3%). También fue mayor entre las personas que requirieron hospitalización por COVID-19 (15,2%), que convivían con alguien que dio positivo en la prueba de la enfermedad (5,4%), que presentaron síntomas graves que requirieron hospitalización (9,8%) y que habían requerido atención de los servicios de salud tres o más veces en el último año (5,4%).

La suspensión también fue más frecuente entre quienes recibieron seguimiento médico durante la pandemia (6,4%), especialmente en los grupos con problemas vasculares (10,1%), gastrointestinales (7,8%) y de salud mental (7,7%).

Aún en el contexto de la salud mental, se observaron mayores tasas de prevalencia en la suspensión de la matrícula entre los estudiantes que reportaron no sentirse bien psicológicamente en el último año (5,0%), que buscaron atención psicológica antes de la pandemia con reportes de diagnósticos de TB (17,0%) y depresión (7,2%) y que recibieron atención psicológica antes de la pandemia (5,5%) y ya recibían atención para tratar la esquizofrenia antes de la pandemia (33,3%).

Además, la suspensión también se observó con mayor frecuencia entre quienes se sometieron a seguimiento psicológico durante la pandemia (6,6%), con diagnósticos de TEPT (17,4%), TAB (17,0%) y SP (10,5%), que tenían un familiar diagnosticado con un trastorno mental (5,8%), especialmente hijos (14,1%) y cónyuges (8,6%); entre quienes ya tenían diagnósticos de un trastorno mental (8,5%), especialmente TEPT (16,2%), TAB (12,9%) y depresión (9,9%); entre quienes necesitaron alejarse de sus actividades debido a que no se sentían bien (8,7%); y entre aquellos con un TMC positivo (5,9%), entre quienes usaron psicofármacos antes de la pandemia (7,5%) y quienes comenzaron a usarlos durante ese período (10,6%). Algunos ejemplos de los psicofármacos más comúnmente usados incluyeron sedantes (11,5%), antipsicóticos (10,7%) y potenciadores cognitivos (10,2%).

El modelo final del análisis de regresión logística reveló los factores de riesgo y protección de los estudiantes de posgrado que solicitaron la suspensión de la matrícula del curso de posgrado *stricto sensu* (Tabla 2).

**Tabla 2-:** Modelo final de regresión logística de factores asociados a la suspensión de un curso de postgrado *stricto sensu* (N = 5.286). Ribeirão Preto, SP, Brasil, 2022

**Variables**	b*	**SE-** b ^**†**^	**Valor Z ^‡^**	p ^§^	**OR ^||^**	**CI** **(95%)** ^¶^	
*(Intercept)*	-4,99	0,32	-15,67	p<0,0010			
Región Nordeste	-0,11	0,28	-0,40	0,69	0,90	0,52	1,54
Región Norte	0,69	0,36	1,93	0,05	2,00	0,96	3,97
Región Sudeste	0,17	0,22	0,78	0,44	1,19	0,78	1,84
Región Sur	-0,03	0,31	-0,08	0,94	0,98	0,52	1,78
Número de hijos/uno	1,03	0,19	5,33	p<0,0010	2,81	1,91	4,08
Número de hijos/dos o más	0,60	0,24	2,49	0,013	1,83	1,12	2,90
Ingeniería	0,75	0,24	3,14	0,0017	2,11	1,30	3,31
Ser becario	-0,69	0,16	-4,23	p<0,0010	0,50	0,36	0,69
Percepción de perjuicios en la formación	0,65	0,20	3,26	0,0011	1,91	1,31	2,85
Diagnóstico autoinformado de trastorno mental	0,88	0,16	5,40	p<0,0010	2,41	1,76	3,33
Necesidad de retirarse de las actividades	1,24	0,18	6,95	p<0,0010	3,44	2,44	4,91
Positivo en TMC**	0,31	0,21	1,50	0,13	1,36	0,92	2,07

*b = Coeficiente de regresión no estandarizado; ^†^SE-b = Error estandarizado; ^‡^Valor Z = Coeficiente de regresión estandarizado; ^§^p = Probabilidad asociada a la prueba estadística; ^||^OR = *Odds Ratio*; ^¶^IC = Intervalo de confianza; **TMC = Trastorno mental común

## Discusión

Los resultados de este estudio indican que las prórrogas y suspensiones de matrículas en programas de postgrado *stricto sensu* en Brasil se asociaron directamente con desafíos académicos, financieros y de salud mental durante el período investigado. La pandemia de COVID-19 exacerbó estos factores al exponer vulnerabilidades estructurales en la educación superior^(20)^ y obstaculizar la continuidad de estudios en diversas áreas del conocimiento^(6-7,11-12,21-23)^. La crisis sanitaria impuso transformaciones abruptas en las instituciones académicas, al requerir adaptaciones en los modelos de enseñanza y el apoyo estudiantil.

Entre los principales factores de riesgo relacionados con las prórrogas de plazo se encontraban estar matriculado en un doctorado, estudiar Ingeniería o Ciencias Biológicas, percibir un perjuicio para la formación y presentar problemas de salud mental. La suspensión de la matrícula, a su vez, se asoció con estar matriculado en un curso de postgrado en Ingeniería, percibir un perjuicio para la formación intensificado por la pandemia, ser diagnosticado con un trastorno mental y necesitar una licencia académica.

Así, si bien la prórroga refleja un intento de mantener la continuidad académica a pesar de la adversidad, la suspensión representa una interrupción más significativa en la trayectoria educativa, generalmente asociada a deterioros emocionales, institucionales o de salud más graves. Si bien ambos resultados están vinculados a la vulnerabilidad estudiantil, requieren respuestas institucionales específicas, adaptadas al grado de deterioro y a las características del contexto en que viven los estudiantes.

La rápida transición al aprendizaje remoto reveló importantes desafíos estructurales, como la falta de infraestructura tecnológica y la brecha digital entre los estudiantes con menor poder adquisitivo^(3,7,14)^. Estos obstáculos han afectado a los programas de postgrado de manera desigual y son particularmente evidentes en campos que dependen de actividades prácticas, como la Ingeniería, donde la transición al aprendizaje remoto ha limitado significativamente el desarrollo de la investigación experimental, especialmente la que requiere acceso a laboratorios y trabajo de campo^(24)^.

Incluso antes de la pandemia, los estudiantes de doctorado ya enfrentaban dificultades para mantener su bienestar físico y mental ante la intensa carga académica y la falta de tiempo para el autocuidado^(9,15,25)^. Durante la pandemia, estos desafíos se vieron agravados por la necesidad de compaginar el aprendizaje remoto, la investigación continua y las responsabilidades familiares, a menudo sin el apoyo financiero o social adecuado^(11-12,26-27)^.

La sobrecarga académica, la incertidumbre sobre el futuro y las pérdidas personales han intensificado trastornos como la ansiedad y la depresión, lo que resalta la necesidad de un apoyo institucional más eficaz^(5,15,28)^. Las altas exigencias académicas y la presión por la productividad han afectado significativamente la salud mental de los estudiantes de postgrado, quienes han experimentado altos niveles de estrés, lo que ha comprometido su retención y rendimiento académico^(5,11)^. Además, la dificultad para equilibrar las exigencias académicas, personales y financieras puede reconocerse como un obstáculo crítico para el bienestar de estos estudiantes y refuerza la importancia de las estrategias institucionales para mitigar estos impactos^(6,11-12)^.

Cabe destacar que la pandemia agravó significativamente el escenario de distrés psicológico entre los estudiantes, especialmente aquellos en situaciones socioeconómicamente vulnerables, debido al aislamiento social y la reducción de las interacciones presenciales^(3-4,7,24)^. La prevalencia de trastornos mentales comunes (TMC) entre los estudiantes brasileños de postgrado alcanzó el 63,5%, asociada a factores como dificultades académicas, inseguridad financiera, extensión de plazos y ausencias temporales de los estudios^(5,27)^. En respuesta a las presiones experimentadas, muchos recurrieron al uso de psicofármacos sin supervisión médica, lo que resalta la urgente necesidad de implementar políticas institucionales que fortalezcan el apoyo a la salud mental y fomenten estrategias de afrontamiento más seguras y efectivas^(9,15,25)^.

Los estudios indican que, durante la pandemia de COVID-19, hubo un aumento significativo en los síntomas de ansiedad y depresión entre los estudiantes universitarios, especialmente entre aquellos vinculados al campo de la salud. La prevalencia de síntomas depresivos osciló entre el 31% y el 38%, mientras que los síntomas de ansiedad alcanzaron aproximadamente el 41%^(29)^. Entre los investigadores de postgrado, aproximadamente el 70% reportó angustia psicológica de leve a severa, a pesar de los niveles más altos de alfabetización en salud mental^(30)^. Estos hallazgos resaltan la importancia de las políticas institucionales para garantizar la atención continua de la salud mental para esta población, particularmente si se considera exposición prolongada a múltiples estresores académicos y personales^(25,27,29-30)^.

La presencia de dificultades académicas intensificadas por la pandemia, la migración a la docencia remota y la sobrecarga experimentada por docentes y estudiantes durante este período pueden haber contribuido a una mayor vulnerabilidad emocional entre los estudiantes, destacando la necesidad de estrategias institucionales para fortalecer el apoyo académico y la salud mental^(11,24)^. Según investigaciones, una relación positiva entre asesores y estudiantes de postgrado puede reducir significativamente el estrés académico (β = -0,27, p < 0,01), con este efecto parcialmente mediado por la autoeficacia (β = -0,14, p < 0,01) y moderado por la resiliencia psicológica (β = -0,10, p < 0,01)^(26)^. En este contexto, la calidad de la asesoría académica y la existencia de un entorno de apoyo son esenciales para minimizar los impactos negativos de los estudios de postgrado^(11,26)^ y reducir la necesidad de prórrogas y suspensiones^(9,12)^.

Entre los participantes que reportaron acceso a clases remotas, se observó una menor frecuencia de extensiones de cursos, lo que explica por qué esto actuó como un factor protector en este subgrupo, aun cuando representara solo el 40% de la muestra. A pesar de las dificultades para adaptarse al aprendizaje remoto, la participación en clases virtuales resultó ser un factor protector contra las extensiones de plazos, posiblemente debido a la flexibilidad que ofrece este modelo. La capacidad de gestionar mejor el tiempo, acceder a nuevas oportunidades de aprendizaje y aumentar la productividad favoreció la continuidad académica de los estudiantes^(6,21-22,24)^.

Estudios previos ya han demostrado que la eliminación de la necesidad de desplazamientos físicos ha facilitado la permanencia de muchos estudiantes de postgrado en sus cursos, al reducir las barreras logísticas y optimizar el tiempo dedicado a las actividades académicas^(4,24)^. Además, la ampliación del acceso a cursos y contenidos que antes estaban restringidos a ciertas ubicaciones, junto con la flexibilidad horaria, ha beneficiado especialmente a los estudiantes que combinan actividades académicas y profesionales, haciendo que la educación sea más accesible y adaptable a sus realidades^(11,24,26)^.

Un hallazgo destacable es que un diagnóstico previo de hipertensión arterial sistémica, aunque clasificado como factor de riesgo para complicaciones por COVID-19, se identificó en este estudio como un factor protector contra la extensión de cursos en programas de postgrado *stricto sensu.* Una posible explicación de este resultado es que los estudiantes con hipertensión, al estar bajo seguimiento médico continuo, tienden a mostrar mayor adherencia a las prácticas de autocuidado, mayor conciencia del manejo del estrés y rutinas personales y académicas mejor estructuradas, incluso ante las adversidades impuestas por la pandemia.

Aunque la hipertensión generalmente se asocia con peores resultados clínicos^(31)^, también puede servir como un marcador para una mayor vigilancia de la salud, particularmente en personas con acceso a servicios médicos y apoyo terapéutico^(32)^. Según estudios recientes, factores como la actividad física, la resiliencia psicológica y las estrategias de afrontamiento positivas pueden mitigar los efectos del estrés en la salud mental y académica de las personas con hipertensión^(31-32)^. Por lo tanto, un diagnóstico previo puede reflejar no solo una condición crónica, sino también una trayectoria de adaptación y atención, lo que puede haber favorecido la continuidad de las actividades académicas durante la pandemia.

Aun así, este es un hallazgo inusual y poco explorado en la literatura científica. Por lo tanto, se requieren más investigaciones en estudios futuros para dilucidar los mecanismos implicados y evaluar si este patrón se confirma en otras poblaciones académicas o contextos institucionales.

Las implicaciones de este estudio refuerzan la urgente necesidad de políticas institucionales orientadas a promover la salud mental y mitigar los impactos de la sobrecarga académica en los programas de postgrado. Entre las estrategias efectivas se incluyen la ampliación del apoyo psicológico, la formación de orientadores, el aumento de la flexibilidad académica y el fortalecimiento de la asistencia estudiantil para garantizar condiciones más equitativas para fomentar la continuidad estudiantil^(5,8-9)^. Asimismo, se destaca la importancia de reformular los marcos curriculares para hacerlos más efectivos y flexibles, garantizando así la sostenibilidad de las carreras de postgrado y reduciendo la incidencia de extensiones y deserciones^(21)^.

El fortalecimiento del apoyo psicológico ha resultado esencial, lo que implica la ampliación de los servicios universitarios, creación de grupos de apoyo, actividades de bienvenida y programas de mentoría académica. Estas iniciativas pueden contribuir a un entorno universitario más saludable y reducir los impactos negativos de la presión académica en los estudiantes^(21,25)^.

Aunque la relación orientador-alumno no se evaluó directamente en este estudio, la literatura destaca su importancia como factor determinante del éxito académico^(26-27)^. Por lo tanto, la formación de tutores es fundamental. Las estrategias institucionales que fomentan la formación continuada de los docentes, con una orientación más humanizada y sensible a las necesidades de los estudiantes, pueden minimizar los impactos negativos de un seguimiento inadecuado. La implementación de directrices institucionales que establezcan las mejores prácticas en orientación académica contribuye directamente a un entorno más productivo, colaborativo y menos estresante^(26-27)^.

Se debe fomentar la flexibilidad académica para permitir ajustes en los plazos de finalización de cursos, disertaciones y tesis, de modo que los estudiantes puedan superar períodos de mayor dificultad sin comprometer su trayectoria académica. Adaptar las evaluaciones y promover currículos más flexibles hace que la educación sea más accesible y sostenible, reduciendo así las tasas de prórrogas de plazos y suspensiones de matrículas^(28)^.

El apoyo financiero y la asistencia estudiantil son esenciales para garantizar la continuidad de los estudiantes de postgrado. La ampliación de las becas de investigación, la asistencia financiera para transporte y alojamiento, y la creación de programas de financiación estudiantil pueden minimizar las tasas de deserción académica por dificultades económicas. Se ha demostrado que la seguridad financiera reduce la vulnerabilidad de los estudiantes y mejora su capacidad para afrontar los desafíos que imponen los estudios de postgrado^(5,21)^.

Además, se debe fomentar el bienestar y las estrategias de afrontamiento saludables mediante actividades culturales, deportivas y sociales, que actúan como factores protectores de la salud mental. Los programas de mentoría entre pares, el fomento de la actividad física y la creación de espacios comunitarios en las universidades ayudan a reducir el estrés y a fortalecer las redes de apoyo social^(26,28)^.

Finalmente, se debe implementar un monitoreo continuo de la salud mental estudiantil mediante encuestas institucionales periódicas para apoyar el desarrollo de políticas de apoyo efectivas. El desarrollo de plataformas anónimas para denunciar la mala conducta académica y el acoso puede contribuir a un entorno académico más seguro y acogedor. La adopción de estrategias institucionales que prioricen tanto la excelencia académica como el bienestar estudiantil es esencial para un sistema educativo más sostenible y equitativo^(3,7,9,11,25,29-30)^.

A pesar de las contribuciones de este estudio, se deben considerar algunas limitaciones. El diseño transversal impide determinar la causalidad entre los factores analizados y los resultados académicos, limitándose a la identificación de asociaciones. La recopilación de datos autoinformados puede haber generado sesgo de respuesta, especialmente en temas sensibles como la salud mental y la inseguridad financiera. Además, la falta de datos cualitativos limita una comprensión más profunda de las percepciones de los estudiantes de postgrado sobre el apoyo institucional y las estrategias individuales de afrontamiento.

La representatividad de la muestra podría no abarcar todas las realidades de la educación de postgrado brasileña, lo que indica la necesidad de realizar futuros estudios que consideren diferentes contextos institucionales y regionales. Se requieren investigaciones longitudinales para evaluar el impacto de las políticas institucionales en la retención académica y la salud mental de los estudiantes a lo largo del tiempo. Además, los enfoques cualitativos pueden profundizar el análisis de los desafíos que enfrentan y las estrategias que utilizan los discentes para afrontar las adversidades académicas^(25)^.

Ante los desafíos identificados, es fundamental que las instituciones educativas inviertan en estrategias integrales que promuevan la salud mental y fortalezcan la retención de estudiantes de postgrado. La ampliación del apoyo psicológico, la formación personalizada de asesores, la flexibilidad académica, la inversión en apoyo estudiantil y la promoción del bienestar deben conformar un conjunto de acciones continuas y accesibles, adaptadas a la diversidad sociocultural y digital del estudiantado. Estas medidas no solo reducen las tasas de deserción escolar y el malestar psicológico, sino que también fortalecen el sistema educativo, por contribuir a una trayectoria académica más equitativa y saludable, propicia para el desarrollo científico y profesional.

## Conclusión

Este estudio demostró que las prórrogas y suspensiones en los cursos de postgrado *stricto sensu* durante la pandemia de COVID-19 se vieron influenciadas por factores de riesgo y protección académicos, personales y psicosociales. Las prórrogas se asociaron principalmente con las inscripciones en programas de doctorado y con carreras orientadas a la Ingeniería y/o Ciencias Biológicas, la percepción de deficiencias en la formación y la presencia de trastornos mentales. Por el contrario, la participación en clases remotas y el diagnóstico previo de hipertensión actuaron como factores de protección, aunque limitaciones como la infraestructura tecnológica deficiente y la reducida interacción social plantearon desafíos significativos para muchos estudiantes.

La suspensión se relacionó con factores como la parentalidad, las dificultades experimentadas durante la pandemia, el historial de ausentismo y los problemas de salud mental, mientras que recibir una beca resultó ser un factor protector. Los resultados destacan la importancia de la salud mental para la retención académica y refuerzan la necesidad de políticas institucionales centradas en el apoyo psicosocial, la reducción de las desigualdades socioeconómicas, la flexibilización del recorrido educativo y la promoción de entornos académicos más inclusivos, especialmente en tiempos de crisis.

## Data Availability

El conjunto de datos que respalda los resultados de este estudio no está disponible públicamente.
